# Transparent, Reproducible Text-Based Phenotyping of Lumbar Intervertebral Disc Degeneration from 500 Consecutive MRI Reports, with a Pre-Specified Image Analysis Framework

**DOI:** 10.3390/diagnostics16182951

**Published:** 2026-09-12

**Authors:** Ahmed Ibrahim Haidar, Mohammed Emam, Abdulwahab Ali Aljubran, Khudhair Mohammed Alkhudhair, Mosa Mohammed Alassiri, Basim Sallah Almutairi, Saleh Abdullah Alsulaiman, Faisal Ibrahim Altamimi, Mashael Mubarak Alkahtani, Ibrahim Ahmed Alyami, Khadijah Mohammed Mobaraki

**Affiliations:** 1 MRI Unit, Radiology Department, Imam Abdulrahman Al Faisal Hospital, First Health Cluster, Riyadh 14723, Saudi Arabia; aihaider@moh.gov.sa (A.I.H.); salsuliman1@moh.gov.sa (S.A.A.); fialtamimi@moh.gov.sa (F.I.A.); 2 Department of Radiologic Technology, College of Applied Medical Sciences, Qassim University, Buraidah 51452, Saudi Arabia; 3 Physics Department, Faculty of Science, Al-Azhar University, Nasr City, Cairo 11884, Egypt; 4 MRI Unit, Radiology Department, Buraidah Central Hospital, Qassim Health Cluster, Buraidah 52361, Saudi Arabia; abaljubran@moh.gov.sa; 5 MRI Unit, Radiology Department, King Saud Medical City (KSMC), First Health Cluster, Riyadh 12746, Saudi Arabia; kalkhudhair@rfhc.gov.sa (K.M.A.); mmassiri@rfhc.gov.sa (M.M.A.); almutairibasim@gmail.com (B.S.A.); ibrahimy78@hotmail.com (I.A.A.); 6 Department of Adult Neurology and Epilepsy, Imam Abdulrahman Al Faisal Hospital, First Health Cluster, Riyadh 14723, Saudi Arabia; mamalkahtani@moh.gov.sa; 7 Al-Amal Medical Complex, Jazan 86622, Saudi Arabia; khadijhmobarki@gmail.com

**Keywords:** lumbar spine, intervertebral disc degeneration, radiology reports, natural language processing, Pfirrmann grading, imaging biomarkers, radiomics, reproducibility, Modic changes, validation

## Abstract

**Background/Objectives:** To characterize the degenerative vocabulary of 500 consecutive lumbar MRI reports using a transparent, reproducible text extraction pipeline; to quantify which descriptors distinguish included from excluded reports and which factors predict text-derived severity; and to specify a reproducible image analysis pipeline whose formal validation against radiologist Pfirrmann grading is defined as the next step. **Methods:** We analyzed 500 lumbar MRI reports (386 patients; December 2018–November 2025), extracting morphological and severity keywords, segmental levels (L1–L2 to L5–S1), Modic mentions, and a custom text-derived ordinal severity (0–3). Keyword prevalence differences were tested with the Fisher exact test under Benjamini–Hochberg false discovery rate control, with 95% confidence intervals (CIs) for all odds ratios (ORs) and risk differences; predictors of severity were examined by ordinal logistic regression. Robustness to within-patient clustering was assessed with patient-clustered robust estimation and one-report-per-patient analyses. A MATLAB pipeline derived per-disc candidate imaging features (predicted Pfirrmann grade, normalized disc height, T2 signal index, quality control) across 316 studies. **Results:** In total, 385/500 reports (77.0%) met the inclusion criteria. The structured morphological keyword field was missing in 49.4% of records, varying by year (58.6% in 2020, 68.7% in 2023, 41.9% in 2024, 33.5% in 2025), indicating systematic reporting drift. Bulge was the dominant descriptor (53.8%); L4–L5 and L5–S1 dominated level mentions. After correction, bulge (OR 6.70, 95% CI 3.86–11.65), mild (5.08, 2.39–10.78), dehydration (9.82, 2.36–40.87) and central (8.63, 2.07–36.01) were enriched among the included reports. All four remained significant in clustering-aware sensitivity analyses. Older age independently predicted higher severity (OR 1.36 per 10 years, 95% CI 1.19–1.56). The image pipeline produced per-disc candidate biomarkers across 316 studies. **Conclusions:** Free-text lumbar MRI reports encode a recognizable but heterogeneous degenerative vocabulary, sufficient for cohort construction yet inconsistent for quantitative grading; the text-derived severity is a noisy proxy. The reproducible image analysis pipeline yields candidate biomarkers whose formal validation against an adjudicated radiologist Pfirrmann reference standard is the explicit, pre-specified next step.

## 1. Introduction

Low back pain is the single largest contributor to years lived with disability worldwide, affecting an estimated 619 million people in 2020 and projected to exceed 800 million prevalent cases by 2050 [1]. Even after three decades of declining age-standardized rates, the absolute burden continues to rise with population growth and aging [1,2]. Magnetic resonance imaging (MRI) is the reference modality for evaluating the degenerative lumbar spine, because it depicts disc hydration, height, herniation, endplate (Modic) change and canal or foraminal compromise without ionizing radiation. However, degenerative findings are highly prevalent in asymptomatic individuals as well, and their association with symptoms is graded rather than categorical [3,4]. This weak and level-dependent imaging–symptom coupling is precisely why the standardized, reproducible phenotyping of the lumbar spine remains an unmet need.

The Pfirrmann classification is the most widely used grading system for disc degeneration on T2-weighted images, and independent studies confirm substantial-to-excellent inter- and intra-observer agreement when it is applied with a defined algorithm [5,6]. Refinements such as the modified eight-grade Pfirrmann scheme and recent morphometric systems improve discrimination, particularly in the elderly or severely degenerated discs, but they remain qualitative and reader-dependent [7,8]. Consequently, two complementary lines of work have emerged. The first applies natural language processing (NLP) to the radiology report itself, converting an unstructured narrative into structured, mineable variables for epidemiology, quality assurance and cohort building [9,10,11]. The second applies deep learning directly to the images, automating vertebral segmentation, disc localization and Pfirrmann or stenosis grading at scale [12,13,14,15,16,17].

Both lines have characteristic failure modes that bear directly on the present study. Report-based NLP inherits the variability of human dictation: the same disc may be called a “bulge”, a “protrusion” or “desiccated”; severity adjectives are applied inconsistently; and not every level is enumerated in every report [9,10]. External validation of report-based models routinely shows a performance drop, underscoring that report language is an imperfect reference standard [11]. Image-based models, conversely, are sensitive to the acquisition protocol, scanner and reconstruction, which is the same reproducibility problem documented extensively in the radiomics literature and addressed through standardization and harmonization [18,19,20,21,22]. A defensible phenotyping workflow must therefore make its extraction rules explicit, quantify its own data completeness and validate text-derived labels against an image-based reference under transparent reporting standards [23,24].

Structured-reporting data mining has been shown to yield valid epidemiological description in other domains, such as urolithiasis, when the underlying fields are consistently populated [25]. Whether free-text lumbar MRI reports, as they exist in routine archives, carry sufficient and sufficiently consistent degenerative signals to support both descriptive epidemiology and downstream image validation has not been systematically described for a consecutive single-center cohort.

Beyond visual assessment, radiomics converts routine images into high-throughput quantitative descriptors of tissue morphology, signal and heterogeneity, yielding mineable imaging biomarkers with diagnostic and prognostic value [26]. Two points from recent syntheses motivate our design. First, a disciplined workflow (dataset definition; acquisition and preprocessing; segmentation; feature extraction and selection; and validation) is a precondition for reproducible quantitative imaging [26]. Second, quantitative features are most informative when integrated with clinical and textual information rather than used in isolation, including in musculoskeletal imaging, while standardization, reproducibility and external validation remain the principal barriers to clinical translation [27]. The pre-specified image analysis framework in this study is therefore designed as the quantitative complement to the report-derived phenotypes, with harmonization and reference-standard validation defined in advance.

We therefore pursued three aims. The first was to describe the documented spectrum of degenerative lumbar disease across 500 consecutive MRI reports using a transparent, rule-based extraction pipeline, reporting cohort accounting, field completeness, keyword prevalence, segmental involvement and a derived ordinal severity at both the report and patient levels. Second, as a pre-specified audit of the selection rule, we sought to test which descriptors could distinguish reports retained for degenerative analysis from those excluded, using the Fisher exact test with false discovery rate control. The third was to specify—in full and reproducibly—an image-based validation framework, including a sequence diagram of the analysis code, so that the text-derived labels could be benchmarked against radiologist Pfirrmann grading in subsequent work. We frame the imaging pipeline explicitly as a proposed and not-yet-executed validation step, with expected performance ranges drawn from the external validation literature rather than from our own images.

To make the evidentiary scope explicit, this study claims (i) the transparent, reproducible text-based phenotyping of 500 consecutive lumbar MRI reports; (ii) a locked, fully published extraction dictionary and severity proxy with audited robustness, including clustering-aware sensitivity analyses; and (iii) an executed, fully automatic image analysis pipeline whose outputs are candidate quantitative features accompanied by a pre-specified validation protocol. It does not claim a validated image-based grading system, agreement with Pfirrmann grading or the diagnostic performance of any imaging feature; these questions define the pre-specified next study (see Proposed Reference Standard for Future Validation).

## 2. Materials and Methods

### 2.1. Study Design and Data Source

This was a single-center, retrospective, descriptive study of consecutive lumbar spine MRI radiology reports. All imaging examinations and reports originated from a single center (King Saud Medical City, Riyadh, Saudi Arabia), whose institutional review board approved the study; “single-center” therefore refers to the source of the data, while the author team is multi-institutional, reflecting an analysis collaboration rather than multiple data sources. The source dataset comprised 500 extracted report rows corresponding to 386 unique patients, with report dates spanning 24 December 2018 to 25 November 2025. The unit of analysis was the report (one row = one report); patient-level summaries were produced by aggregation where indicated. Reporting follows the STROBE framework for observational studies; the prospective elements of the proposed imaging sub-study are aligned to the Checklist for Artificial Intelligence in Medical Imaging (CLAIM) and to general guidance on the design and reporting of model-versus-clinician comparisons [23,24]. Because the pre-specified validation stage will develop and evaluate models linking imaging features to ordinal grades, its reporting will additionally follow the TRIPOD+AI statement [28], complementing the STROBE and CLAIM items already applied.

**Cohort accounting.** The 500 consecutive report rows (24 December 2018 to 25 November 2025) correspond to 386 unique patients: 313 patients contributed one report, 48 contributed two reports, 9 contributed three reports and 16 contributed four reports; repeated examinations therefore account for 187 of 500 rows (37.4%). Applying the eligibility rule left 385 included reports from 295 patients; 115 reports were excluded. By verbatim reason, the exclusions comprised trauma or fracture (54), previous lumbar surgery (35), infection (8), combined indications (prior surgery with trauma or infection, 7), another non-degenerative or non-lumbar primary diagnosis (8) and normal studies (3); the deterministic priority rule of Section 2.2 maps these to the four primary categories of Section 3.1 (trauma/fracture, 58; prior surgery, 38; infection, 8; non-degenerative/normal, 11). Of the 385 included reports, 316 examinations (82.1%) had complete, retrievable DICOM series in the archive and entered the automated pipeline; the remaining 69 could not be processed because the corresponding series were unavailable or incomplete at retrieval. These denominators (500 reports; 385 included reports; 316 processed studies) are used consistently throughout.

### 2.2. Eligibility Criteria

Reports were eligible (“included”) when the primary documented process was degenerative lumbar disease. Reports were excluded when the dominant finding was trauma or fracture, when the study was post-operative (prior instrumentation or laminectomy), when there was active or prior infection or when the study was normal or described a non-degenerative, non-lumbar or otherwise out-of-scope pathology. Exclusion reasons were captured verbatim and then mapped to four mutually exclusive primary categories by a deterministic priority rule (trauma/fracture > prior surgery > infection > non-degenerative/normal/other). Eligibility was determined solely from the radiologist’s summary diagnosis in the report (whether the primary documented process was degenerative lumbar disease), recorded before and independently of any keyword processing; the keyword dictionary of Section 2.3 was applied afterward, uniformly to all 500 reports. No keyword count or enrichment statistic was used to decide eligibility.

### 2.3. Data Extraction and Variables

From each report, we extracted the following: patient identifier and report date (join keys); age and sex where recorded; a free-text morphological keyword field (benchmark); the source context/level narrative; the primary clinical findings narrative; documented Modic change status; and inclusion/exclusion status. Two families of derived variables were generated by deterministic text rules: (i) segmental involvement—for each level, i.e., L1–L2, L2–L3, L3–L4, L4–L5 and L5–S1, a Boolean indicating explicit mention, plus a per-report count of enumerated levels; (ii) morphological/severity keyword flags (for example, bulge, protrusion, herniation, extrusion, dehydration, stenosis, spondylolisthesis, foraminal, central, facet, Modic and the severity adjectives mild/moderate/severe).

### 2.4. Derived Ordinal Severity (Transparent Rule Set)

To enable a balanced, reproducible severity description, the text from the morphological keyword, level context and primary findings fields was combined and mapped to an ordinal scale via the following published rule set.

1. **Grade 0 (none/normal):** Normal height and signal, no degenerative descriptor.
2. **Grade 1 (mild):** Disc bulge or diffuse bulge.
3. **Grade 2 (moderate):** Protrusion, annular tear/fissure, disc dehydration/desiccation or loss of/reduction in disc height.
4. **Grade 3 (severe):** Extrusion, sequestration, severe/marked degeneration, canal stenosis or nerve root compression.

Where several descriptors co-occurred in one report, the highest applicable grade was assigned (worst-level convention). The mapping is intentionally simple and auditable; as detailed in Section 2.6, it is treated as a candidate label requiring image-based validation, rather than as a clinical ground truth [5,8]. The four ordinal categories follow the morphological hierarchy of the consensus lumbar disc nomenclature (version 2.0) [29] and group descriptors by escalating structural compromise: grade 1, contour abnormality without focal displacement (bulge); grade 2, focal displacement or established degenerative change confined to the disc (protrusion, annular fissure, dehydration/desiccation, disc height loss); grade 3, displacement beyond the disc space or neural compromise (extrusion, sequestration, canal stenosis, nerve root compression) and explicit high-severity qualifiers. The ordering is anchored in consensus nomenclature and clinical actionability rather than fitted to the data; the assignment of individual synonyms to grades involved author judgment and is published in full (dictionary, precedence and negation rules; Supplementary Methods) so that the mapping can be independently audited or re-specified. All severity statistics in this article are computed under this locked rule; its executable specification accompanies the Supplementary Methods, and the analysis code and derived per-report severity file are archived (Data Availability Statement). The score is a text-derived ordinal proxy for reported severity; it is not a clinical grading instrument and is not equivalent to the Pfirrmann classification, which is based on the T2 signal intensity, nucleus–annulus distinction and disc height rather than on lexical descriptors; the reference-standard comparison is pre-specified in the Proposed Reference Standard for Future Validation section.

### 2.5. Statistical Analysis

Descriptive statistics are reported as counts and percentages for categorical variables and as the mean (standard deviation, SD), median and interquartile range (IQR) for continuous variables. Field completeness is expressed as the percentage of missing values per variable. To identify descriptors that differed between included and excluded reports, each keyword was cross-tabulated by inclusion status and tested with the two-sided Fisher exact test [30]. Because fifteen keywords were tested simultaneously, *p*-values were adjusted by the Benjamini–Hochberg procedure, controlling the false discovery rate (FDR) [31]. For an ordered list of *p*-values p(1) ≤ ... ≤ p(m), the largest rank k for which p(k) ≤ (k/m)·α defines the rejection threshold; q-values are the corresponding adjusted *p*-values, and significance was declared at q < 0.05. For each keyword, we additionally report the risk difference (inclusion rate minus exclusion rate) and the odds ratio (OR) with its 95% confidence interval; the four FDR-significant descriptors have intervals that exclude 1. In this manuscript, 95% confidence intervals are reported for every odds ratio and risk difference across all fifteen keywords (Wald intervals on the log odds ratio, with a Haldane–Anscombe 0.5 correction for zero cells; Newcombe intervals for risk differences). To identify predictors of the derived severity, an ordinal (proportional odds) logistic regression was fitted, with age, sex and report year as covariates. The proportional odds (parallel lines) assumption was formally evaluated with a likelihood ratio comparison of the ordered-logit and multinomial-logit models; threshold-specific binary-logit estimates (severity ≥ 1, ≥2, and ≥3) are additionally reported as a descriptive check. Had the assumption been rejected, threshold-specific (partial proportional odds) estimates would have been presented as the primary description of covariate effects.

For the proposed image-based validation (Section 2.6), the pre-specified primary agreement metric is the quadratic-weighted Cohen κ [32] between image-derived and radiologist Pfirrmann grades, defined as κ = 1 − (Σ w_ij_ O_ij_)/(Σ w_ij_ E_ij_), with quadratic weights w_ij_ = (i − j)^2^/(G − 1)^2^ for a G-category scale, O the observed and E the expected (chance) agreement matrices. Secondary metrics are the confusion matrix, the within-one-grade accuracy, the macro-F1, the receiver operating characteristic and precision–recall area under the curve (AUC) for clinically actionable thresholds (moderate-or-worse, severe) and the Spearman ρ between continuous imaging features and the report-derived severity, each with 95% confidence intervals obtained by bootstrap. All analyses were performed in Python 3.11 (Python Software Foundation, Wilmington, DE, USA; descriptive and inferential statistics) and MATLAB R2025b (The MathWorks, Inc., Natick, MA, USA; image feature extraction).

#### Statistical Handling of Repeated Examinations Within Patients

Because the 500 reports arose from 386 patients (73 patients, 18.9%, contributed two to four reports, accounting for 187 of 500 rows, 37.4%), all report-level comparisons were examined for within-patient correlation. Keyword enrichment odds ratios were re-estimated with cluster-robust (sandwich) standard errors clustered on patient, using logistic models with an independence working correlation, with Benjamini–Hochberg correction as in the primary analysis. As an exact sensitivity analysis that removed clustering by design, all Fisher tests were repeated on one report per patient under three rules: the most recent report, the earliest report and 1000 random draws. For the ordinal severity model, we report (i) the report-level proportional odds model with a patient-clustered bootstrap confidence interval (2000 resamples of patients) and (ii) a patient-level model using each patient’s maximum severity. Full results are given in Supplementary Table S1 (clustering sensitivity analyses); the analysis code is archived with the Supplementary Methods.

### 2.6. Image-Based Validation Pipeline and Pending Agreement Analysis

**Figure 1:**
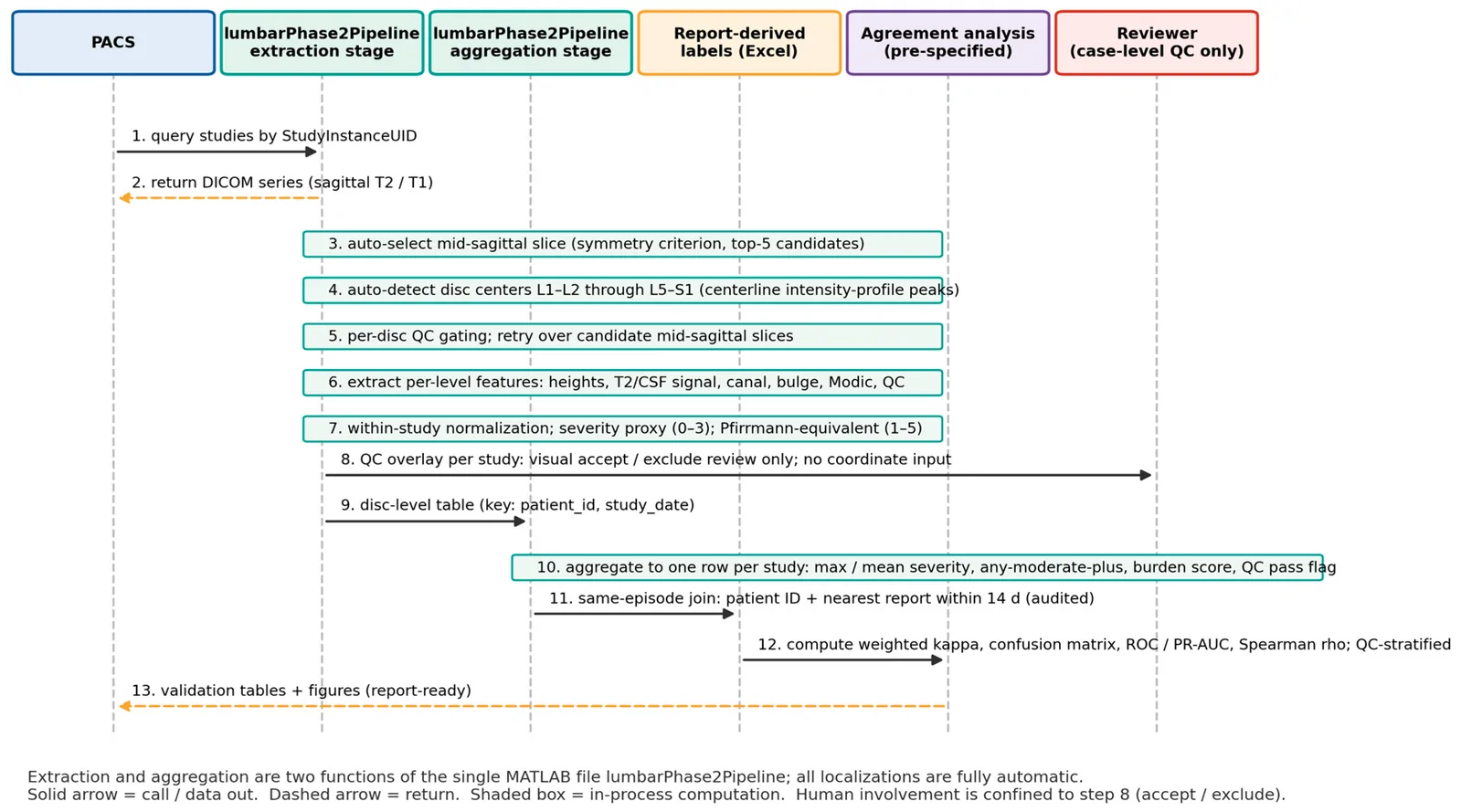
Sequence diagram of the image-based validation pipeline (lumbarPhase2Pipeline, a single function-based MATLAB file, including its study-level aggregation stage), showing DICOM retrieval, automatic mid-sagittal slice selection, automatic disc center detection with the pipeline-generated quality control overlay, per-level feature extraction and within-study normalization, study-level aggregation, the join with report-derived labels and the computation of QC-stratified agreement metrics. All localizations are produced without manual input; the overlay is used only for post hoc, case-level visual quality review (accept/exclude), and no coordinates are edited by the operator.

To move from text-derived labels toward image-based phenotyping, we developed a two-stage MATLAB pipeline that is directly joinable to the report dataset on the patient identifier and same-episode examination date. The first script, *lumbarPhase2Pipeline (a single, function-based MATLAB file)*, reads DICOM studies grouped by StudyInstanceUID, automatically selects the mid-sagittal slice using a symmetry criterion and automatically detects the disc centers L1–L2 through L5–S1 from the spine centerline vertical intensity profile (bright and dark discs by peak prominence), with per-disc quality control gating and a retry over candidate mid-sagittal slices (a fully automatic step, with no manual disc center selection). For each detected level, it extracts the disc height (in pixels and normalized to the adjacent vertebral body height), a T2 signal index referenced to intracanal cerebrospinal fluid, the nucleus–annulus signal ratio, endplate (Modic pattern) signal ratios, the canal anteroposterior diameter and the posterior disc bulge in millimeters (converted through the DICOM pixel spacing) and a per-disc quality control flag; heights and signals are normalized within each study, with cohort-level winsorization and percentile-calibrated candidate flags applied at the merge stage, and each disc receives a heuristic severity proxy (0–3) together with an explicitly heuristic Pfirrmann-equivalent grade (1–5) from fixed T2 signal and height ratio cutoffs; all of these are unvalidated candidate outputs (see Proposed Reference Standard for Future Validation and Section 3.8). The second script, *the same file (study-level aggregation)*, aggregates the disc-level export to one row per study (the maximum and mean severity, an any-moderate-or-worse flag, the minimum/maximum/mean of the normalized height and signal, a continuous degeneration burden score and a study-level QC pass flag). The study-level table is then joined to the report-derived labels on the canonicalized patient identifier under a documented same-episode date rule (an exact scan–report date match is preferred; otherwise, the nearest report within a pre-specified 14-day window is accepted, reflecting the normal interval between acquisition and reporting, and each merged row carries the signed scan–report interval in days and the matching rule used as audit columns), and the agreement metrics of Section 2.5 are computed, with the results stratified by QC status to reflect deployable performance. Figure 1 provides the sequence diagram of this workflow. For clarity, mid-sagittal slice selection, disc center detection and all downstream measurements are fully automatic; no manual disc center selection or coordinate editing is performed at any step. The only human involvement in the imaging pipeline is the case-level quality review of the automatically generated overlays (accept or exclude).

This pipeline was executed across 316 studies to produce the per-disc imaging features and quality control overlays reported in Section 3.8. The formal agreement analysis against an adjudicated per-level Pfirrmann reference standard has not yet been performed; accordingly, the anticipated performance ranges (Section 4) are taken from external validation studies of comparable open models, rather than from our own data, in order to avoid presenting expected values as observed ones [11,14,15].

#### Proposed Reference Standard for Future Validation

For the pre-specified validation (not undertaken in the present study), we propose a stratified random sample of approximately 120–200 discs, stratified by text-derived severity and lumbar level to span the degenerative range, graded per level on the Pfirrmann I–V scale by at least two radiologists working independently and blinded to the pipeline output and to the report-derived labels, with disagreements resolved by consensus or third-reader adjudication and with the inter-reader reliability reported. The adjudicated per-level grade would serve as the reference for the agreement analysis pre-specified in Section 2.5.

### 2.7. Ethics and Data Governance

All direct patient identifiers (names) present in the source extract were removed prior to analysis; only a non-identifying study key (patient code and report date) was retained for the planned image–text join. The study used existing radiology reports and did not alter clinical care. Institutional review board/ethics approval reference and the data availability statement are documented in the Declarations section in accordance with the journal’s policy.

## 3. Results

### 3.1. Cohort Accounting and Completeness

**Figure 2:**
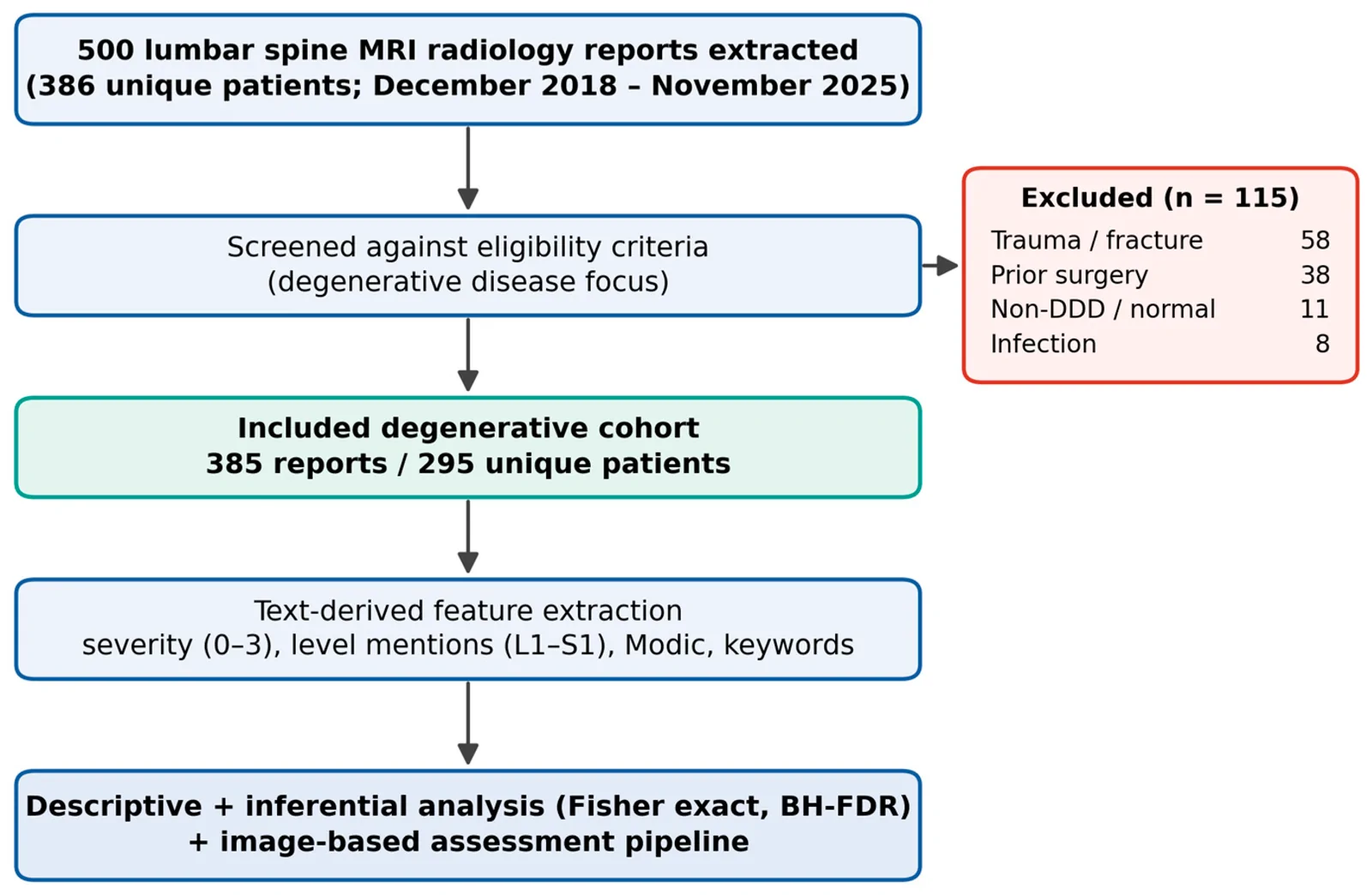
Cohort accounting and analytic workflow, from 500 extracted reports to the included degenerative cohort and the descriptive, inferential and image-based assessment stages.

**Figure 3:**
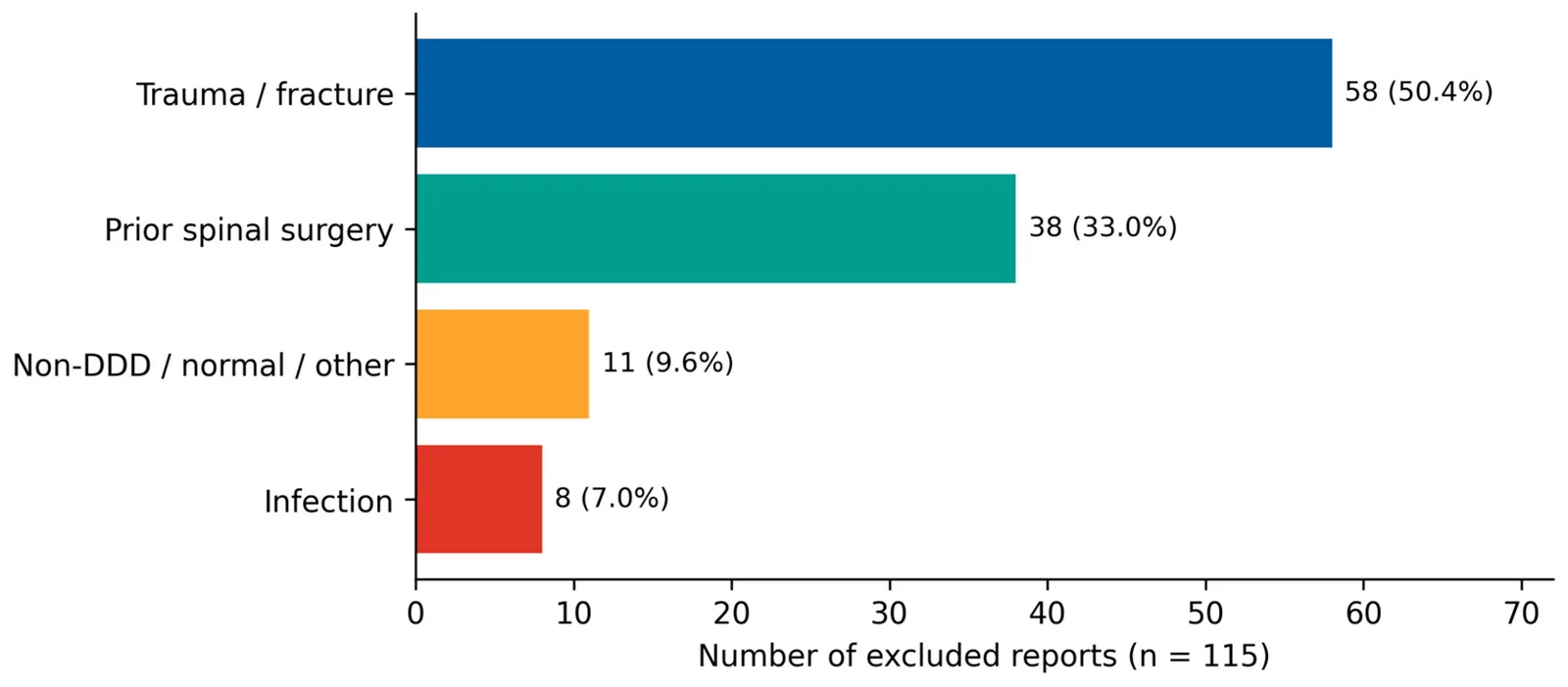
Primary reasons for report exclusion (*n* = 115), mapped to four mutually exclusive categories by a deterministic priority rule.

Of 500 extracted reports, 385 (77.0%) met the degenerative disease inclusion criteria and 115 (23.0%) were excluded (Figure 2, Table 1). The 500 reports represented 386 unique patients; 295 unique patients contributed at least one included report. Most patients contributed a single report (313/386), with 48, 9 and 16 patients contributing two, three and four reports, respectively. Exclusions were dominated by trauma or fracture (58/115, 50.4%) and prior spinal surgery (38/115, 33.0%), followed by non-degenerative/normal studies (11/115, 9.6%) and infection (8/115, 7.0%) (Figure 3). Seven of the 115 reports carried combined indications (prior surgery with trauma or infection) and were assigned by the priority rule (four to trauma/fracture, three to prior surgery); the eleven non-degenerative/normal studies comprised eight with another non-degenerative or non-lumbar primary diagnosis and three normal studies (Section 2.1).

**Figure 4:**
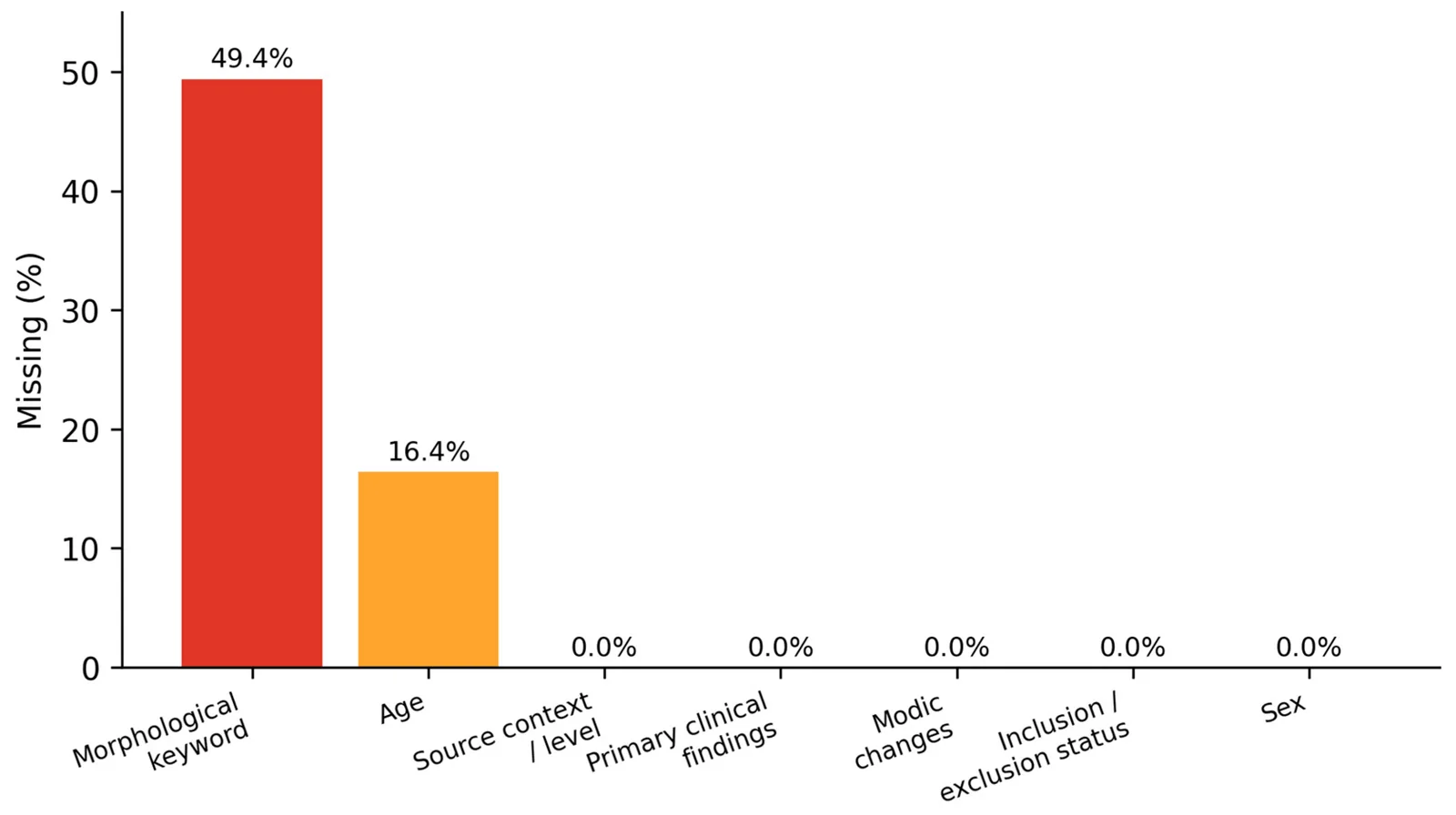
Field completeness across extracted variables. The structured morphological keyword field is missing in 49.4% of records, whereas the narrative and status fields are complete.

The narrative and status fields were complete: source context/level, primary clinical findings, Modic change status and inclusion/exclusion status each had 0% missingness. By contrast, the structured morphological keyword (benchmark) field was missing in 49.4% of records, and a numeric age was unavailable in 16.4% (many age/sex fields contained only “M” or “F”) (Table 2, Figure 4). The dataset is thus strong in terms of narrative content but weak in terms of a consistently populated structured morphology label—the central motivation for downstream standardization and image-based quantification. Analyzed by year, this missingness was not random but followed a non-monotonic, year-dependent pattern, rising from 58.6% in 2020 to 68.7% in 2023 before falling to 41.9% in 2024 and 33.5% in 2025, indicating systematic reporting template drift rather than random loss.

**Figure 5:**
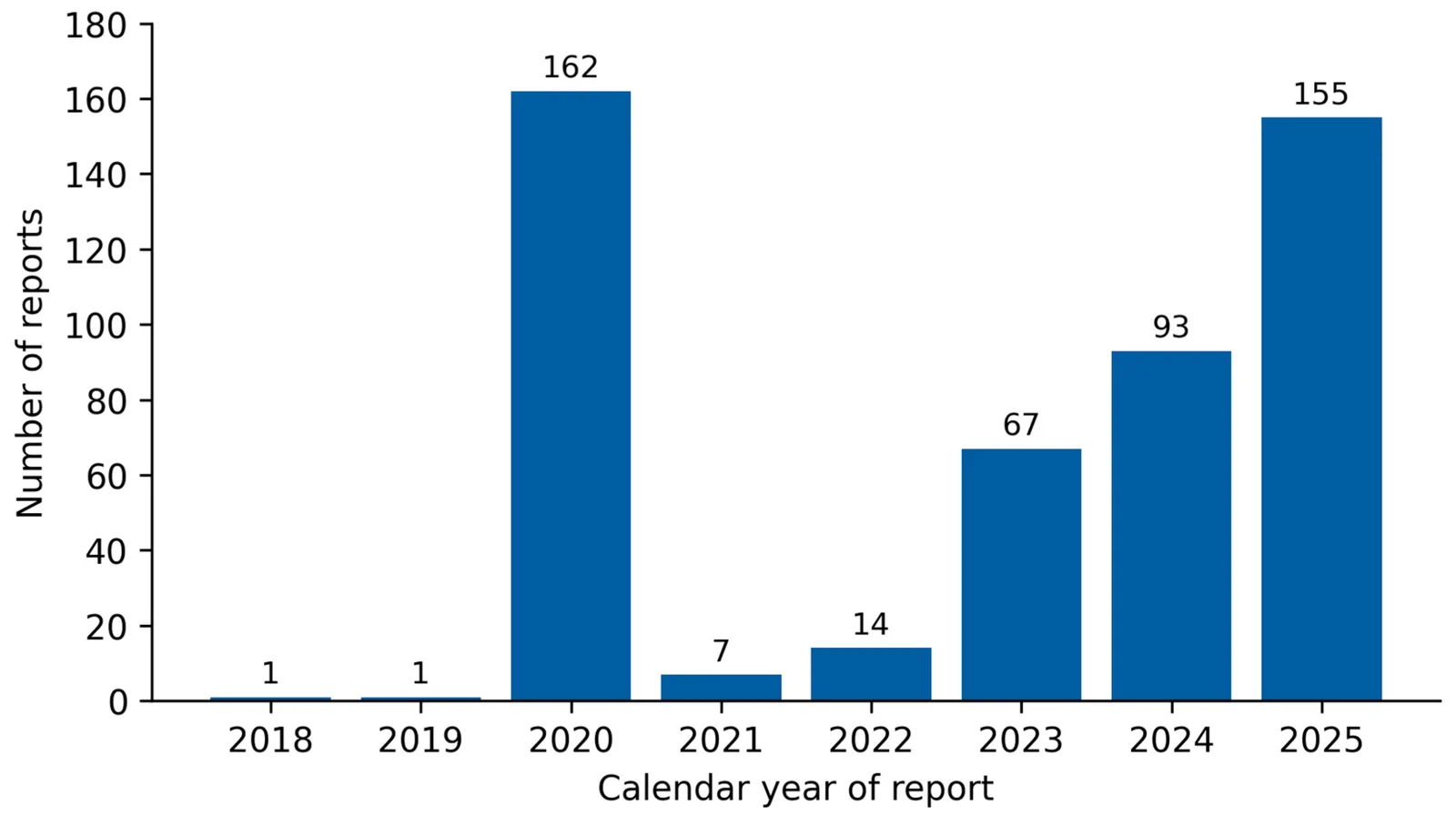
Temporal distribution of reports by calendar year (2018–2025), indicating uneven coverage that should be considered as a potential source of protocol and reporting drift confounding.

Reports were unevenly distributed across the study window, concentrated in 2020 (*n* = 162), 2025 (*n* = 155), 2024 (*n* = 93) and 2023 (*n* = 67) (Figure 5). This temporal heterogeneity is relevant because scanner upgrades, protocol revisions and reporting style drift across years can confound any later modeling, and it motivates the QC-stratified validation described in Section 2.6.

### 3.2. Demographics

**Figure 6:**
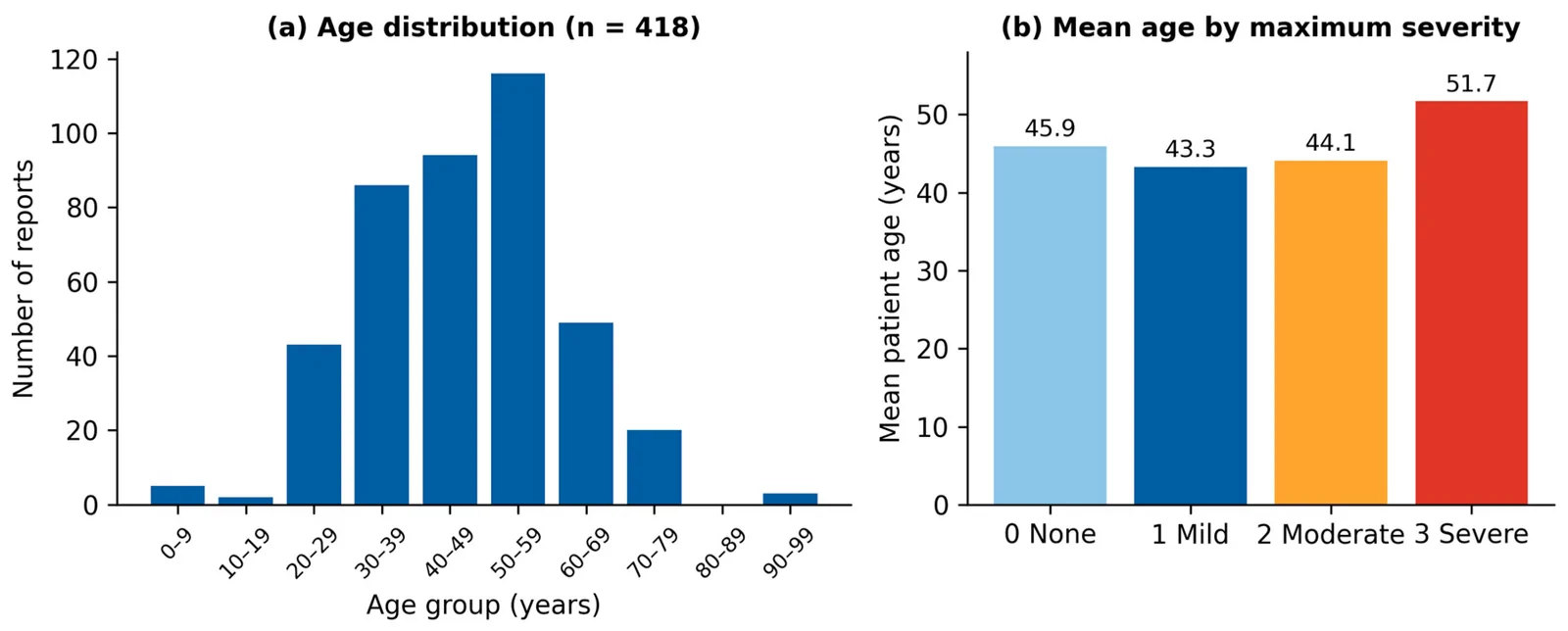
(**a**) Patient age distribution; (**b**) mean patient age by maximum derived severity grade, showing higher mean age in the severe phenotype.

Among reports with a parseable age (*n* = 418), the mean age was 46.7 years (SD 14.5; median 48; IQR 37–56; range up to 93). The report-level sex distribution was 51.8% female and 48.2% male; at the patient level, it was 52.3% female and 47.7% male, consistent with the recognized female preponderance of symptomatic lumbar degeneration [1]. The age distribution peaked in the fifth and sixth decades (Figure 6a).

### 3.3. Prevalence of Text-Derived Descriptors

**Figure 7:**
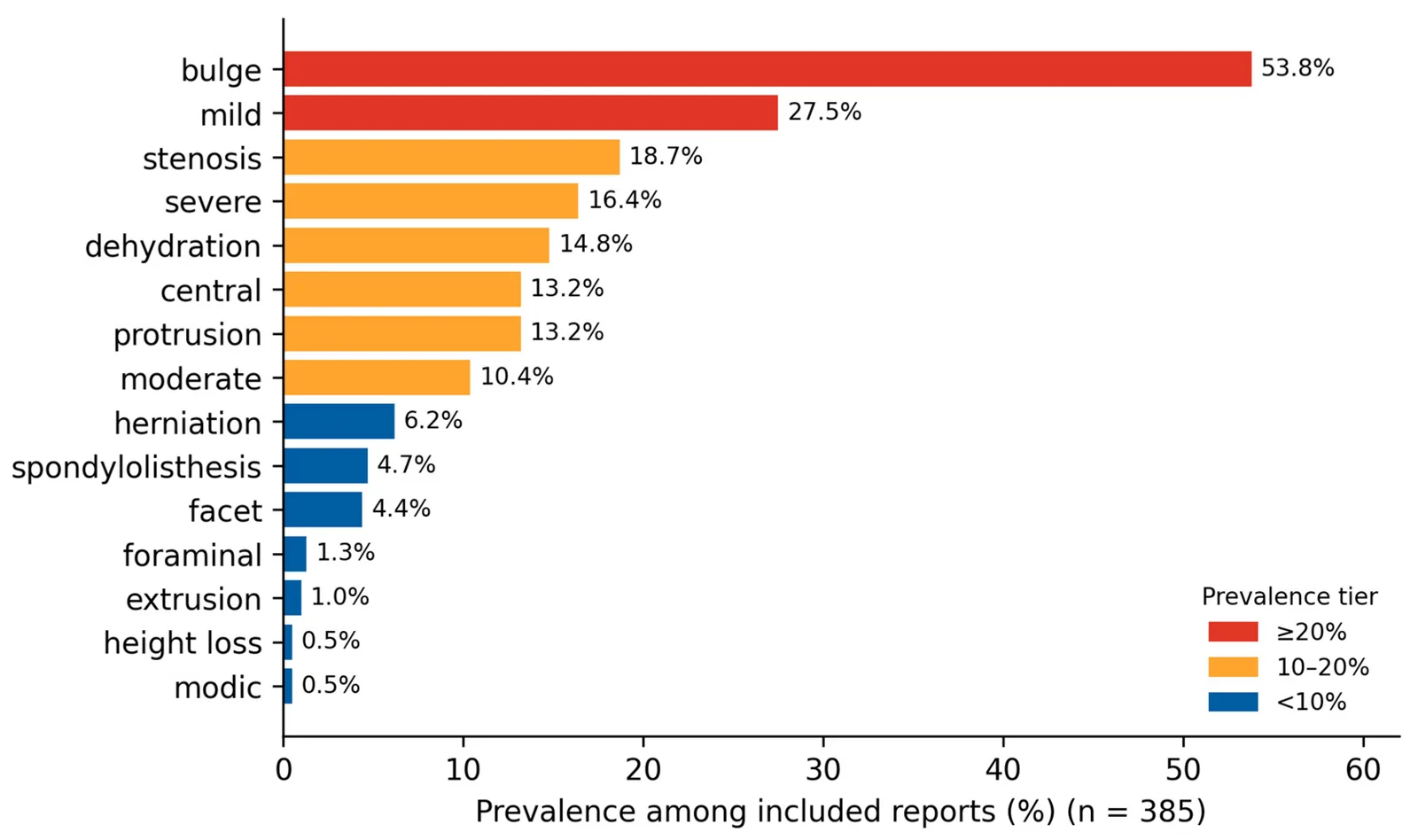
Prevalence of text-derived morphological and severity descriptors among included reports (*n* = 385). Color encodes prevalence tier (≥20%, 10–20%, <10%).

Within the included reports, disc bulge was by far the most frequent descriptor (53.8%), followed by the severity adjective mild (27.5%), stenosis (18.7%), severe (16.4%), dehydration (14.8%), protrusion (13.2%) and central (13.2%) (Table 3, Figure 7). Lower-prevalence terms included moderate (10.4%), herniation (6.2%), spondylolisthesis (4.7%), facet (4.4%), foraminal (1.3%), extrusion (1.0%), and Modic and height loss (0.5% each). The vocabulary therefore mixes morphology, severity and location terms, confirming that the report text contains recognizable degenerative terminology but in a heterogeneous, non-quantitative form.

### 3.4. Selection Criteria Audit: Keyword Enrichment Between Included and Excluded Reports

**Figure 8:**
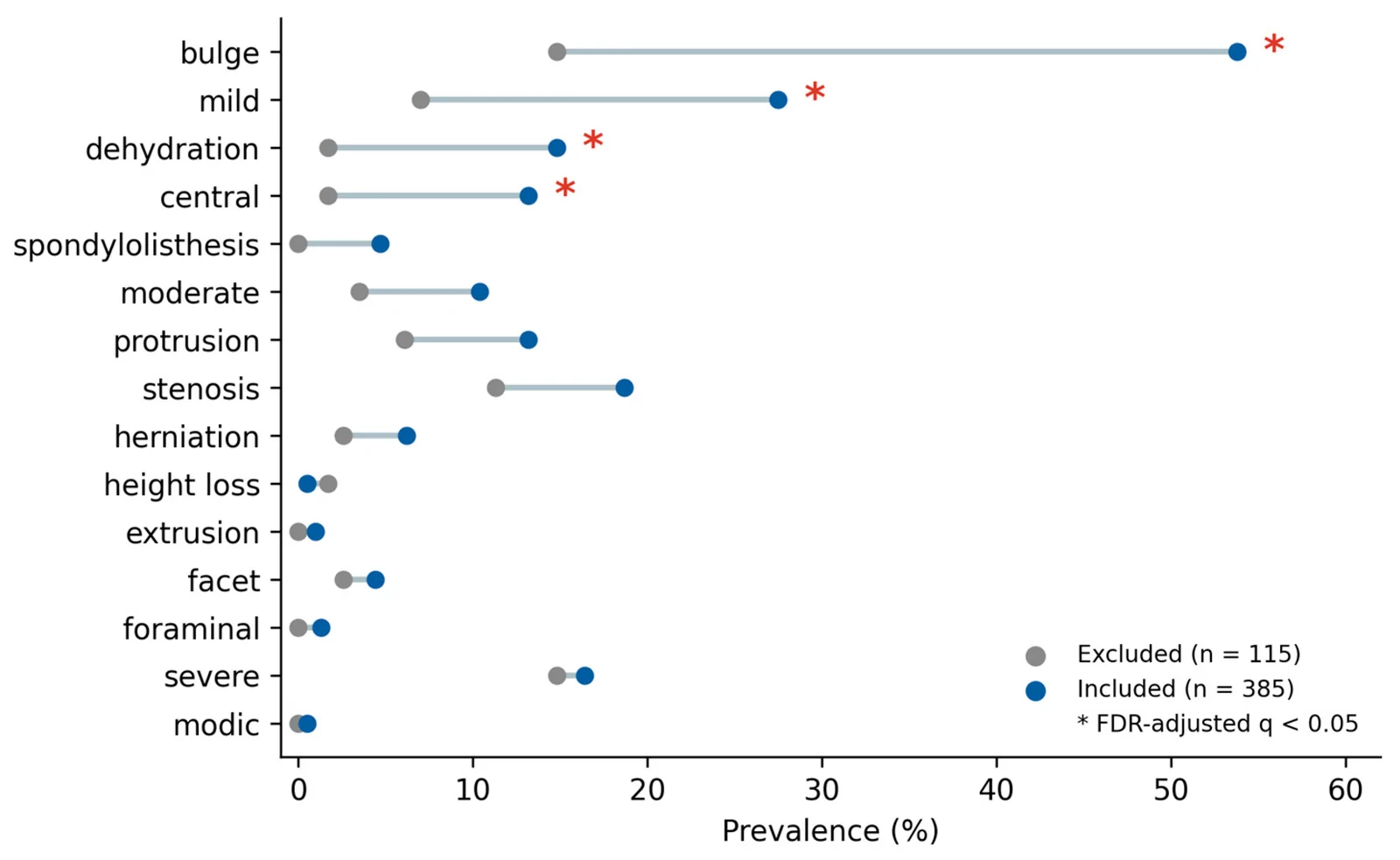
Keyword prevalence in included versus excluded reports. Asterisks denote significance at FDR-adjusted q < 0.05 (Fisher exact test with Benjamini–Hochberg correction).

Because eligibility required that the radiologist’s primary documented process be degenerative lumbar disease, the enrichment of degeneration-related vocabulary among the included reports is expected by construction. This analysis is therefore presented as an audit of the selection rule, and the odds ratios quantify how strongly the report vocabulary tracks the eligibility decision, not an independent biological association. Fisher exact testing with Benjamini–Hochberg FDR control identified four descriptors that were significantly enriched among included relative to excluded reports (Table 3, Figure 8): bulge (53.8% vs. 14.8%; risk difference 39.0 percentage points; OR 6.7; q < 0.001), mild (27.5% vs. 7.0%; OR 5.08; q < 0.001), dehydration (14.8% vs. 1.7%; OR 9.82; q < 0.001) and central (13.2% vs. 1.7%; OR 8.63; q < 0.001). Several descriptors showed nominal but non-significant enrichment after correction, including spondylolisthesis (q = 0.054), moderate (q = 0.058), protrusion (q = 0.097) and stenosis (q = 0.126). The severity adjective “severe” did not differ between groups (16.4% vs. 14.8%; q = 0.83), consistent with severe language appearing in both degenerative and non-degenerative (e.g., traumatic) reports. Inclusion status is therefore not random with respect to the report language, which has implications for selection bias in any text-defined cohort. Inference was unchanged after accounting for within-patient correlation: with patient-clustered robust standard errors, and separately in exact one-report-per-patient analyses (*n* = 386; most recent report, earliest report and 1000 random draws), the same four keywords remained significant after false discovery rate correction (Supplementary Table S1: clustering sensitivity analyses). The cluster-robust odds ratios were 6.70 for bulge (95% CI 3.67–12.26), 5.08 for mild (2.36–10.95), 9.82 for dehydration (2.33–41.36) and 8.63 for central (2.04–36.47), with point estimates unchanged from the primary analysis.

### 3.5. Segmental Involvement

**Figure 9:**
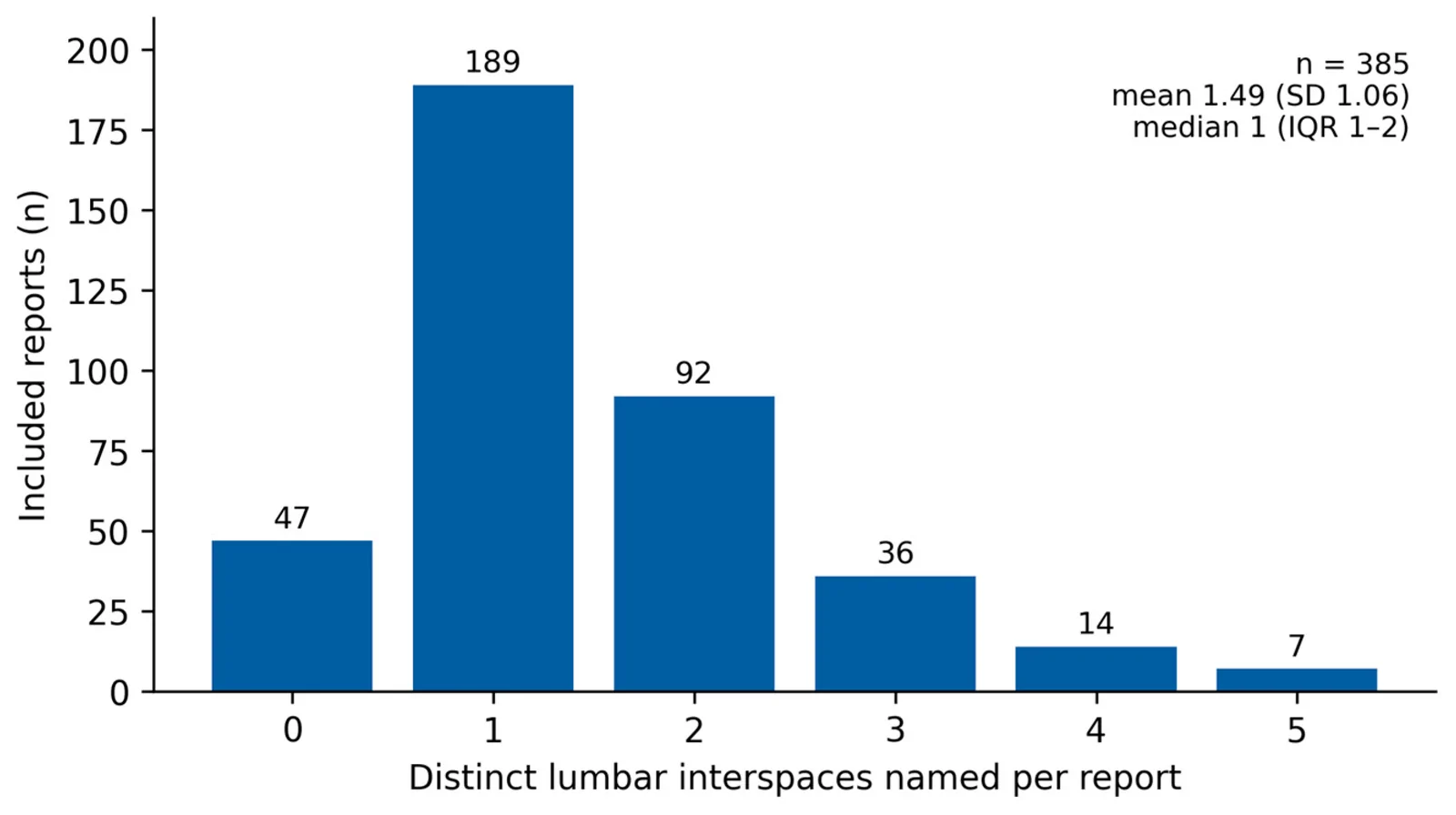
Number of distinct lumbar interspaces named per included report under the locked definition (*n* = 385; mean 1.49, SD 1.06; median 1; range 0–5).

**Figure 10:**
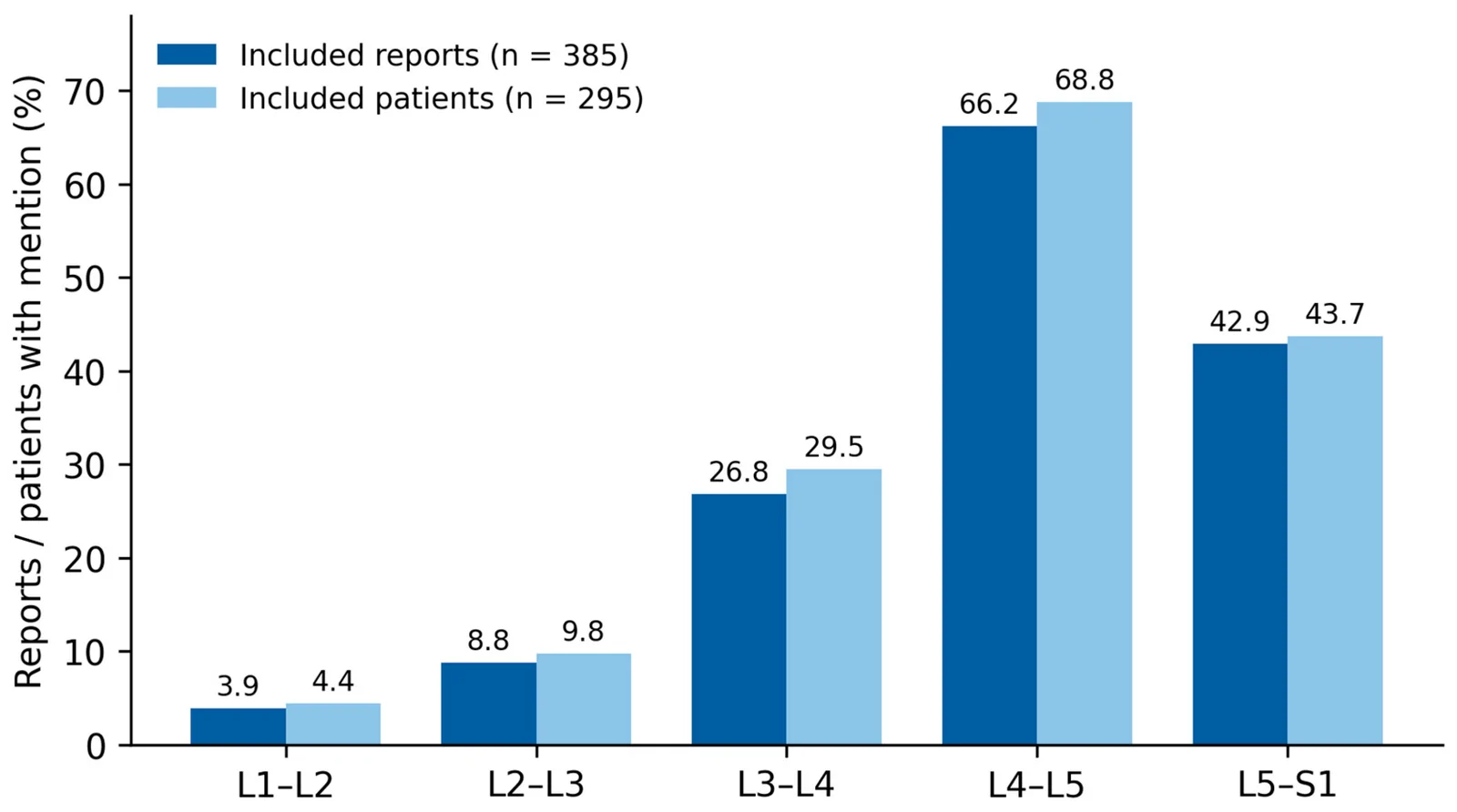
Per-interspace mention prevalence under the locked definition (distinct lumbar interspaces, L1–L2 through L5–S1) among included reports (*n* = 385) and included patients (*n* = 295), showing caudal predominance at L4–L5 and L5–S1.

Under the locked definition adopted in this manuscript, level mentions are counted as distinct named lumbar interspaces (L1–L2 through L5–S1) after deduplication and the expansion of explicit spans, with shorthand forms (for example, L4/5) parsed; the maximum possible value is therefore five. Within the included subset (*n* = 385), reports named a median of one distinct lumbar interspace (IQR 1–2; mean 1.49, SD 1.06; range 0–5). Forty-seven reports (12.2%) named no specific interspace, 189 (49.1%) named one and 149 (38.7%) named two or more (two: 92; three: 36; four: 14; five: 7) (Figure 9). The most frequently named interspaces were L4–L5 (255 reports, 66.2%) and L5–S1 (165 reports, 42.9%), followed by L3–L4 (103, 26.8%), L2–L3 (34, 8.8%) and L1–L2 (15, 3.9%); the patient-level pattern was concordant (Figure 10). This caudal predominance is concordant with the established topography of lumbar degeneration and Modic change [3,4], and the variation in the number of interspaces named reflects heterogeneous reporting granularity rather than the absence of disease. A previously reported token count summary (mean 2.84, maximum 7), which counted repeated level tokens and vertebral body references rather than distinct lumbar interspaces, was internally inconsistent and has been removed; all level statistics in this article use the locked definition above.

### 3.6. Derived Severity Distribution

**Figure 11:**
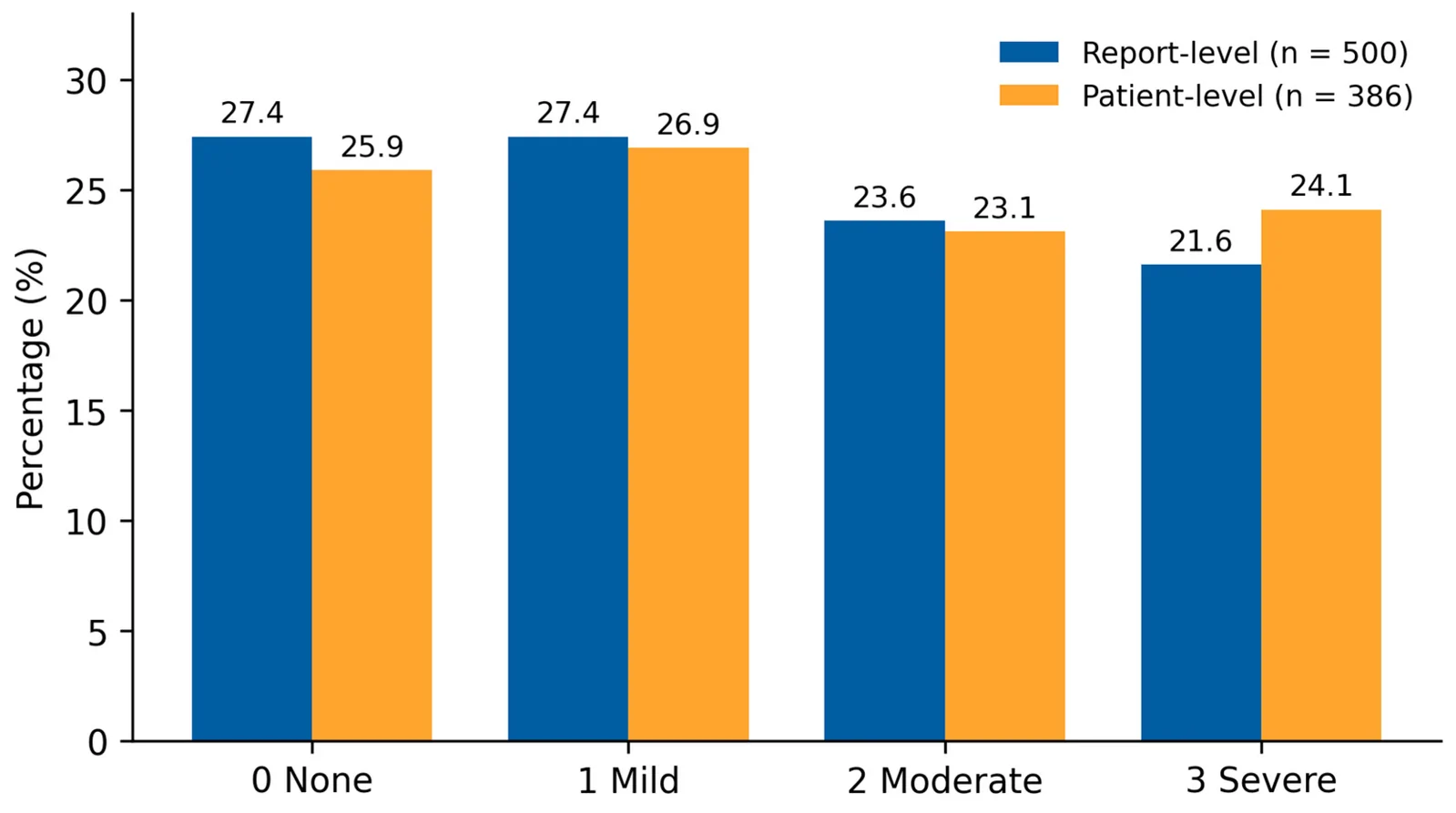
Derived degeneration severity (0–3) at report and patient level, showing a broadly balanced distribution suitable for benchmarking.

The rule-based ordinal severity was reasonably balanced. At the report level (*n* = 500), the distribution was grade 0—27.4% (*n* = 137), grade 1—27.4% (*n* = 137), grade 2—23.6% (*n* = 118) and grade 3—21.6% (*n* = 108) (Table 4). At the patient level (maximum severity per patient, *n* = 386), it was 25.9%—none, 26.9%—mild, 23.1%—moderate and 24.1%—severe (Figure 11). The mean patient age increased with the maximum severity in a clinically plausible, non-monotonic pattern: grade 0, 45.9 years; grade 1, 43.3 years; grade 2, 44.1 years; grade 3, 51.7 years. This was consistent with the highest-grade (severe) phenotype being associated with older age (Figure 6b). The balance of the derived scale makes it suitable as a candidate label for the image-based validation described below, while its reliance on report wording is the reason that validation is required. In an ordinal logistic regression (*n* = 417 reports with a parseable, non-zero age; of the 418 parseable ages in Section 3.2, one report recorded an implausible age of 0 and was treated as missing), older age independently predicted higher derived severity (OR 1.36 per 10 years, 95% CI 1.19–1.56; *p* < 0.001), whereas female sex (OR 0.97, 95% CI 0.68–1.38) and report year (OR 1.00 per year, 95% CI 0.91–1.09) were not significant. The age association was robust to within-patient correlation: the report-level odds ratio per decade of 1.36 carried a patient-clustered bootstrap 95% CI of 1.14–1.65 (2000 patient resamples, percentile method), and the patient-level model (*n* = 340 patients with a parseable age; maximum severity per patient) gave an OR per decade of 1.24 (95% CI 1.07–1.44). The parallel-lines likelihood ratio test comparing the ordered-logit and multinomial-logit models gave a chi-square of 42.83 (df = 6; *p* < 0.001), rejecting the proportional odds assumption; per the pre-specified rule (Section 2.5), the threshold-specific binary-logit age odds ratios are therefore presented alongside the primary model and rose monotonically with the severity (≥1: 1.13, 95% CI 0.97–1.32; ≥2: 1.30, 1.13–1.51; ≥3: 1.70, 1.42–2.05; Supplementary Table S1).

### 3.7. Modic Change Documentation

Modic changes were explicitly mentioned in 9.0% of reports (45/500) and in 9.6% of patients (37/386). This documented prevalence is consistent with population-based estimates of MRI Modic change and reinforces the notion that endplate signal change, although clinically relevant, is inconsistently transcribed in routine reports and is a natural target for image-based detection [4].

### 3.8. Image-Based Pipeline Output and Quality Control Across the Cohort

**Figure 12:**
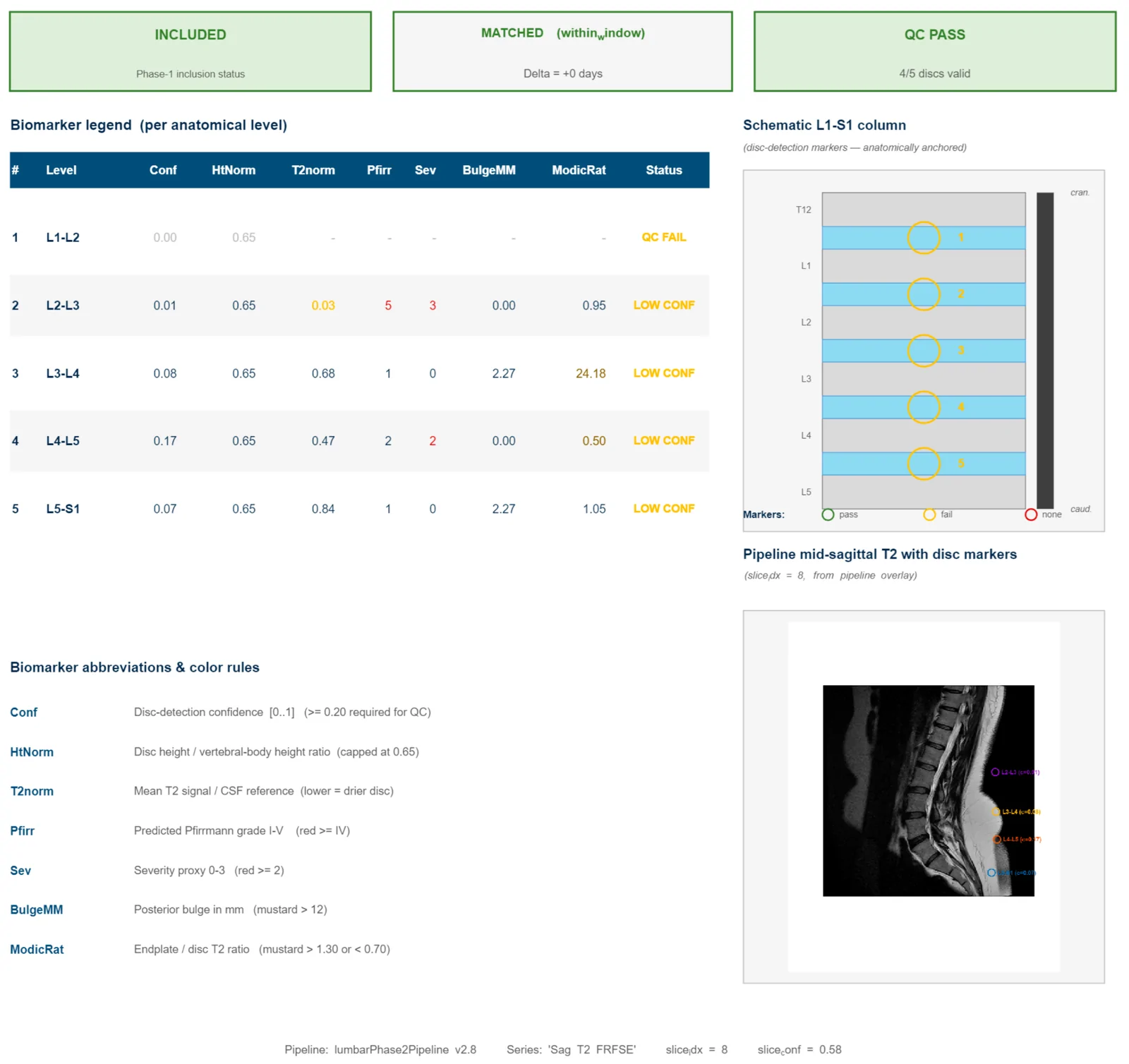
Representative de-identified per-study quality control overlay from the executed image-based pipeline: the inclusion/imaging–report match/QC header, the per-level biomarker table (detection confidence, normalized disc height, T2 signal index, predicted Pfirrmann grade, severity, bulge and Modic ratio), a schematic L1–S1 column and the mid-sagittal T2 with anchored disc markers. Patient identifier and study date were removed for presentation. Outputs shown are candidate quantitative features produced by the automated pipeline for illustration; no comparison against a radiologist reference standard has been performed, and no accuracy claim is made or implied (validation pre-specified in the Proposed Reference Standard for Future Validation section). Color coding: green header fields denote included / matched / QC pass and red denote excluded, unmatched or QC failure; in the status column, red marks a per-level QC failure and amber a low detection confidence. In the biomarker table, red numerals flag a predicted Pfirrmann grade ≥ IV or severity ≥ 2, and mustard numerals flag a posterior bulge > 12 mm or an endplate/disc T2 ratio outside 0.70–1.30, as defined in the in-figure legend.

All quantities in this section are unvalidated candidate biomarkers; their agreement with expert grading is the pre-specified objective of the future validation study (see Proposed Reference Standard for Future Validation and Section 3.9) and is not evaluated here. The image-based pipeline described in Section 2.6 was executed across 316 studies. For each study, it produced a per-disc record (detection confidence, normalized disc height, T2 signal index, predicted Pfirrmann grade, ordinal severity (0–3), bulge magnitude and a Modic ratio), rendered as a per-study quality control (QC) overlay. A representative de-identified overlay is shown in Figure 12: a header summarizing the inclusion status, report–imaging match and QC; a per-level biomarker table; a schematic L1–S1 column; and the mid-sagittal T2 with anchored disc markers. The deployable validation set comprises studies that both pass per-level disc detection QC and are matched to a contemporaneous report within the time window; studies with a per-level QC failure or without a matched report are gated out, so that any reported performance reflects deployable rather than best-case behavior.

**Figure 13:**
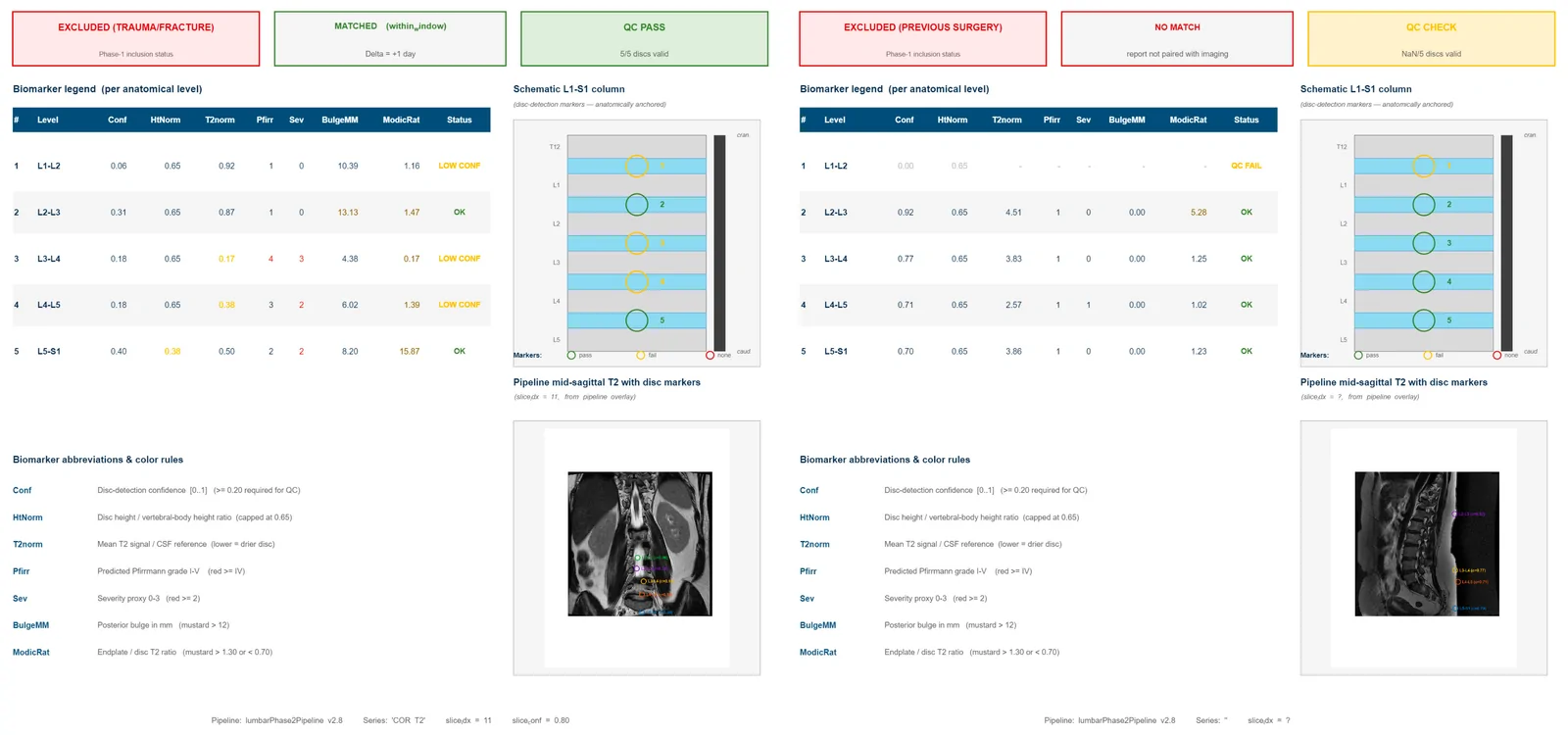
Quality control across the cohort (de-identified examples). (**a**) A study with severe, multilevel degeneration (predicted Pfirrmann IV–V, severity 2–3), showing that the pipeline grades advanced disease; (**b**) a study held out of the deployable validation set because it fails a per-level QC check and lacks a matched report. Each of the 316 rendered studies was summarized by such an overlay. Outputs shown are candidate quantitative features produced by the automated pipeline for illustration; no comparison against a radiologist reference standard has been performed, and no accuracy claim is made or implied (validation pre-specified in the Proposed Reference Standard for Future Validation section). Color coding follows Figure 12 (green = included / matched / QC pass; red = excluded, unmatched or QC failure; amber = low confidence or QC check; red and mustard numerals = threshold flags defined in the in-figure legend).

Across the cohort, the overlays span the full degenerative range and make the gating that defines the validation set auditable (Figure 13). The pipeline assigns high predicted Pfirrmann grades and severities to studies with marked, multilevel degeneration (Figure 13a), confirming that it grades advanced disease rather than only normal discs; conversely, a study that fails a per-level QC check or cannot be paired with a contemporaneous report is held out of the deployable validation set (Figure 13b). Presenting QC- and match-stratified outputs in this way, rather than a single pooled figure, follows the QC-stratified reporting principle adopted throughout (Section 2.6) and keeps the exclusion logic transparent.

These per-disc outputs are the direct input to the agreement analysis pre-specified in Section 2.5. The remaining step is to compute this agreement against an adjudicated, per-level Pfirrmann reference standard; because this gold set is not yet available, no agreement statistic is reported here. Pre-specified expectations, taken from external validation studies of comparable open models rather than from our own data, are summarized in Table 5 [11,14,15]. Throughout this article, these image-derived outputs are described as candidate imaging biomarkers; the framework under which their formal agreement with an expert reference standard will be quantified is pre-specified in Section 3.9.

### 3.9. Pre-Specified Image-Based Validation Framework (Future Work)

The formal agreement analysis between the image-derived candidate grades and an adjudicated per-level Pfirrmann reference standard has not been performed and is not claimed here; it is fully pre-specified as the immediate next step. The reference standard and reader/adjudication procedure are defined in the Proposed Reference Standard for Future Validation section, and the agreement metrics, quadratic-weighted Cohen κ, confusion matrix, within-one-grade accuracy, macro-F1, ROC and precision–recall AUC for moderate-or-worse and severe thresholds, calibration and Spearman ρ for continuous features, each with bootstrap 95% CIs and stratified by quality control, are pre-specified in Section 2.5. Until this reference standard is available, the per-disc outputs of Section 3.8 are reported as candidate imaging biomarkers only.

## 4. Discussion

In a consecutive single-center series of 500 lumbar MRI reports, free-text radiology narratives encoded a recognizable degenerative vocabulary dominated by disc bulge and caudal (L4–L5, L5–S1) involvement that was sufficient to construct a degenerative cohort (385/500) and to derive a balanced ordinal severity, yet it was simultaneously heterogeneous, incompletely structured (morphological keyword field missing in 49.4%) and variable in the comprehensiveness of level enumeration. These two observations signal sufficiency for cohorting but insufficiency for grading and define the central message and the rationale for image-based validation.

The descriptive findings align with the wider literature. The dominance of bulge and the caudal concentration of degeneration reproduce well-established topographic patterns, and the documented Modic prevalence (9–10%) is of the same order as in population-based MRI estimates [3,4]. The female preponderance and the rise in mean age for the severe phenotype are likewise concordant with epidemiological data on lumbar degeneration and its disability burden [1,2]. The fact that bulge, mild, dehydration and central were the descriptors that were most enriched in the included versus excluded reports is methodologically important: it demonstrates quantitatively that a text-defined degenerative cohort is a selected sample with respect to language—a selection effect that must be acknowledged whenever report text is used as a filter or label [10,11].

Viewed through a radiomics and imaging AI lens, the limiting factor is reproducibility rather than the availability of a signal. The same concerns that the Image Biomarker Standardization Initiative and the reproducibility literature raise for quantitative features—sensitivity to acquisition, segmentation and processing—apply, in a linguistic form, to report-derived labels: inconsistent vocabulary is the textual analog of unharmonized feature extraction [18,19]. Two methodological perspectives are therefore essential and are sometimes omitted in report mining studies. The first is harmonization: multi-scanner, multi-protocol and multi-year data such as ours (Figure 5) require the explicit harmonization of any downstream imaging features, for which ComBat and its variants are the most validated approach [20,21,22]. The second is segmentation and reference-standard variability: because Pfirrmann grading is itself reader-dependent, the validation reference should be a consensus, adjudicated, per-level grade rather than a single read [5,6,8]. Our proposed pipeline addresses both by exporting QC flags for stratified analysis and by specifying per-level consensus labeling.

The proposed validation framework is deliberately conservative. Rather than reporting image-derived agreement, which we did not measure, we anchor our expectations to external validation evidence: open models such as SpineNet achieve a weighted κ on the order of 0.65–0.85 against radiologist Pfirrmann grading and balanced accuracy near 78% for disc degeneration and 86% for Modic detection, with the agreement falling when the reference is a noisy report-derived label rather than image-based grading [14,15]. Independent comparisons report fair-to-substantial—not perfect—concordance and emphasize ongoing refinement [15]. Realistic targets when validating image-derived outputs against our text-derived severity are therefore moderate: a weighted κ of roughly 0.35–0.60 and AUC of roughly 0.70–0.85 for the moderate-or-worse threshold, improving under QC filtering. Stating these as expectations, explicitly distinguished from observed results, is consistent with reporting standards that caution against overclaiming model performance [23,24].

The strengths of this work are the transparent and auditable extraction rules, the dual report- and patient-level accounting, the inferential treatment of keyword enrichment with FDR control rather than uncorrected testing and the full, reproducible specification of the validation code (Figure 1). These choices respond directly to documented weaknesses in imaging AI and report mining studies, namely underreported data handling and absent external validation [11,24].

**Relation to prior work.** Two established lines bound this study: image-only automated grading (e.g., SpineNet and its external validations [14,15]; deep learning stenosis/Pfirrmann models [12,13,17]) and NLP-based extraction from radiology reports [9,10,11,33,34]. Our contribution is not a new grading network but a transparent, governable bridge between them: fully auditable rule-based extraction; an explicit text-to-image join on patient and date keys with a pre-specified agreement protocol; quality control-stratified deployable reporting; and end-to-end reproducibility through a published rule set, pipeline scripts and sequence diagram. Where large, curated deep learning models remain stronger in raw grading accuracy, this workflow adds transparency, data governance and hybrid linkage suited to routine archive deployment.

**Clinical implications.** The pipeline can standardize and auto-populate the structured morphology field that is absent in 49.4% of reports; enable large, mineable degeneration cohorts for epidemiology and audit; reduce inter-reporter language variability by attaching a reproducible, QC-gated severity index; and flag moderate-or-worse discs for reader attention as a triage aid. Each of these represents an advancement beyond free-text-only reporting.

## 5. Limitations

Several limitations follow from the data and design and should temper interpretation. First, the analysis is report-based: the labels reflect components that radiologists chose to dictate and not a direct image assessment, so absent terms denote non-mention rather than confirmed absence, and the derived severity is a noisy proxy that has not yet been validated against images. Second, the source is a single center with uneven temporal coverage (Figure 5), limiting generalizability and introducing possible protocol and reporting drift effects. Third, the structured morphological keyword field was missing in 49.4% of records and numeric age was missing in 16.4%, so the prevalence estimates for some descriptors are conservative, and age analyses are restricted to reports with parseable ages. Fourth, keyword detection is lexical and may be affected by negation, synonymy and abbreviation; we did not deploy a transformer-based negation-aware extractor, which prior work shows can change the measured prevalence [9,10]. Fifth, although the imaging pipeline was executed for per-disc feature extraction and quality control across 316 studies, the formal agreement analysis against an adjudicated per-level Pfirrmann gold standard has not yet been performed; the quoted performance ranges are therefore external expectations and not measured results. Sixth, no clinical outcomes (pain, function, surgery) were linked, so the clinical meaning of the derived phenotypes is not established. Finally, the heuristic 0–3 mapping, although transparent, collapses morphology and severity into a single ordinal axis and will not capture every clinically relevant distinction [7,8]. In addition, the imaging outputs reported here are single-center candidate features whose agreement with an expert reference standard has not yet been measured; this will require both the pre-specified validation described in the Proposed Reference Standard for Future Validation section and multicenter confirmation. The imaging pipeline is fully automatic, with human involvement confined to the case-level quality review of the generated overlays. Moreover, although the morphological keyword missingness is shown here to be systematic across years rather than random, it still renders some prevalence estimates conservative. Incorporation bias is inherent to the included-versus-excluded keyword comparison: the same clinical construct that defined eligibility also generates the vocabulary being compared. We mitigated the interpretive risk by presenting the analysis as a selection criteria audit and by avoiding etiological language, but the reported enrichment should not be read as an independent association.

## 6. Recommendations and Future Directions

- **Build an adjudicated, per-level imaging reference standard.** Sample 100–200 studies stratified by derived severity and obtain consensus Pfirrmann grades per disc from at least two readers with disagreement adjudication, rather than relying on report-derived labels [5,6,8].
- **Execute the specified MATLAB pipeline and validate against this reference.** Report the pre-specified quadratic-weighted Cohen κ with bootstrap 95% confidence intervals, the confusion matrix, the within-one-grade accuracy, the ROC/PR-AUC for moderate-or-worse and severe thresholds and the Spearman ρ for continuous features, all stratified by QC status to reflect deployable performance [14,15].
- **Harmonize before pooling.** Given multi-year, potentially multi-scanner acquisition, apply ComBat or a validated variant to any imaging features prior to statistical comparison and report feature stability [18,19,20,21,22].
- **Upgrade the text extractor.** Replace lexical matching with a negation- and context-aware transformer/LLM extractor and quantify the change in measured prevalence and inter-method agreement against the lexical baseline [9,10,11,34].
- **Pursue external and multi-center validation.** Replicate in an independent center and, where possible, benchmark against an open model such as SpineNet to position performance against published external validation ranges [11,14,15].
- **Report to standard and link outcomes.** Complete a CLAIM checklist for the imaging sub-study, register the analysis and link image-derived phenotypes to clinical outcomes to establish clinical validity [23,24].

## 7. Conclusions

Across 500 consecutive lumbar MRI reports, free-text narratives carried a recognizable but heterogeneous degenerative vocabulary, dominated by disc bulge and caudal (L4–L5, L5–S1) involvement, that supported reproducible cohort construction and a balanced, rule-based severity description while remaining inconsistent in structure and granularity. Text-derived labels are therefore adequate for descriptive epidemiology and cohort building but should be treated as a noisy proxy for grading. Older age independently predicted higher derived severity, and the structured morphology field showed systematic, year-dependent missingness. The reproducible image analysis pipeline yields per-disc candidate imaging biomarkers whose formal validation against an adjudicated radiologist Pfirrmann reference standard is the explicit, pre-specified next step, together with multicenter external validation, feature harmonization and outcome linkage.

## Figures and Tables

**Table 1:** Cohort accounting and temporal coverage.

Metric	Value
Reports extracted (total)	500
Included (degenerative)	385 (77.0%)
Excluded	115 (23.0%)
Unique patients (total)	386
Unique patients (≥1 included report)	295
Reports per patient (1/2/3/4)	313/48/9/16
Report date range	24 December 2018–25 November 2025

**Table 2:** Field completeness (missingness) for extracted variables.

Field	Missing (%)
Morphological keyword (benchmark)	49.4
Age (numeric)	16.4
Source context/level	0
Primary clinical findings	0
Modic change status	0
Inclusion/exclusion status	0
Sex	0

**Table 3:** Keyword prevalence and association with inclusion status (Fisher exact test with Benjamini–Hochberg FDR; sorted by risk difference). Here, 95% CIs are included for all ORs and risk differences (OR: Wald with Haldane–Anscombe 0.5 correction for zero cells, marked *; risk difference: Newcombe).

Keyword	Incl. %	Excl. %	Risk Diff. (pp)	OR	q (FDR)
bulge	53.8	14.8	39.0 (29.9 to 46.2)	6.70 (3.86–11.65)	<0.001 *
mild	27.5	7.0	20.6 (13.1 to 26.3)	5.08 (2.39–10.78)	<0.001 *
dehydration	14.8	1.7	13.1 (7.6 to 17.2)	9.82 (2.36–40.87)	<0.001 *
central	13.2	1.7	11.5 (6.2 to 15.5)	8.63 (2.07–36.01)	<0.001 *
stenosis	18.7	11.3	7.4 (−0.5 to 13.6)	1.80 (0.96–3.39)	0.126
protrusion	13.2	6.1	7.2 (0.5 to 12.0)	2.36 (1.04–5.35)	0.097
moderate	10.4	3.5	6.9 (1.1 to 11.0)	3.22 (1.13–9.19)	0.058
spondylolisthesis	4.7	0.0	4.7 (1.0 to 7.3)	11.63 * (0.70–194.47)	0.054
herniation	6.2	2.6	3.6 (−1.6 to 7.0)	2.48 (0.73–8.40)	0.269
severe	16.4	14.8	1.6 (−6.7 to 8.3)	1.13 (0.63–2.02)	0.828

pp, percentage points; OR, odds ratio (indicates undefined OR due to a zero cell); * q < 0.05. The full 15-keyword table is provided in the Supplementary Materials.

**Table 4:** Derived degeneration severity (0–3) at report and patient level.

Severity Grade	Report n	Report %	Patient %
0—None/normal	137	27.4	25.9
1—Mild	137	27.4	26.9
2—Moderate	118	23.6	23.1
3—Severe	108	21.6	24.1

**Table 5:** Pre-specified expectations for the pending image-based validation (external validation literature; not measured in this study).

Scenario	Expected Agreement
Image-based grade vs. radiologist Pfirrmann	Weighted κ ≈ 0.65–0.85
Modic detection (T1/T2 available)	AUC ≈ 0.85–0.95
Image-derived vs. text-derived severity (noisy proxy)	Weighted κ ≈ 0.35–0.60
Moderate-or-worse threshold	AUC ≈ 0.70–0.85

## Data Availability

The de-identified derived data supporting the findings of this study (extracted keyword counts, severity and segmental-level distributions, and statistical outputs) are contained within the article and its Supplementary Materials. The underlying de-identified per-report and per-study datasets, together with the analysis code (the text extraction rule set and the image processing pipeline for per-disc feature extraction and quality control), are available from the corresponding author (M. Emam; m.emam@qu.edu.sa) on reasonable request, subject to institutional data governance approval and a data use agreement. The raw radiology reports and source MRI studies cannot be shared publicly because they constitute protected medical records.

## Supplementary Materials

The following supporting information can be downloaded at https://www.mdpi.com/article/10.3390/diagnostics16182951/s1; Supplementary Methods S1: Text corpus and preprocessing; S2: Keyword extraction dictionary (Table 3, Figure 7); S3: Text-derived severity proxy (0–3): dictionary, precedence, and negation rules; S4: Locked definition of level mentions (Section 3.5; Figure 8 and Figure 9); S5: Clustering sensitivity analyses (Section Statistical Handling of Repeated Examinations Within Patients; Supplementary Table S1); S6: Image-analysis pipeline: annotated code excerpts (Section 2.6); S7: Code and data archiving; verification statement; Table S1: Clustering sensitivity analyses for the keyword-enrichment comparison between included (*n* = 385) and excluded (*n* = 115) reports.

## References

[1] Ferreira M.L., de Luca K., Haile L.M., Steinmetz J.D., Culbreth G.T., Cross M., Kopec J.A., Ferreira P.H., Blyth F.M., Buchbinder R. (2023). Global, regional, and national burden of low back pain, 1990–2020, its attributable risk factors, and projections to 2050: A systematic analysis of the Global Burden of Disease Study 2021. Lancet Rheumatol..

[2] Hoy D., March L., Brooks P., Blyth F., Woolf A., Bain C., Williams G., Smith E., Vos T., Barendregt J. (2014). The global burden of low back pain: Estimates from the Global Burden of Disease 2010 study. Ann. Rheum. Dis..

[3] Brinjikji W., Diehn F.E., Jarvik J.G., Carr C.M., Kallmes D.F., Murad M.H., Luetmer P.H. (2015). MRI findings of disc degeneration are more prevalent in adults with low back pain than in asymptomatic controls: A systematic review and meta-analysis. AJNR Am. J. Neuroradiol..

[4] Mok F.P.S., Samartzis D., Karppinen J., Fong D.Y.T., Luk K.D.K., Cheung K.M.C. (2016). Modic changes of the lumbar spine: Prevalence, risk factors, and association with disc degeneration and low back pain in a large-scale population-based cohort. Spine J..

[5] Pfirrmann C.W.A., Metzdorf A., Zanetti M., Hodler J., Boos N. (2001). Magnetic resonance classification of lumbar intervertebral disc degeneration. Spine.

[6] Urrutia J., Besa P., Campos M., Cikutovic P., Cabezon M., Molina M., Cruz J.P. (2016). The Pfirrmann classification of lumbar intervertebral disc degeneration: An independent inter- and intra-observer agreement assessment. Eur. Spine J..

[7] Griffith J.F., Wang Y.X.J., Antonio G.E., Choi K.C., Yu A., Ahuja A.T., Leung P.C. (2007). Modified Pfirrmann grading system for lumbar intervertebral disc degeneration. Spine.

[8] Soydan Z., Bayramoglu E., Urut D.U., Iplikcioglu A.C., Sen C. (2024). Tracing the disc: The novel qualitative morphometric MRI based disc degeneration classification system. JOR Spine.

[9] Sorin V., Barash Y., Konen E., Klang E. (2020). Deep learning for natural language processing in radiology—Fundamentals and a systematic review. J. Am. Coll. Radiol..

[10] Dada A., Ufer T.L., Kim M., Hasin M., Spieker N., Forsting M., Nensa F., Egger J., Kleesiek J. (2024). Information extraction from weakly structured radiological reports with natural language queries. Eur. Radiol..

[11] Reichenpfader D., Müller H., Denecke K. (2024). A scoping review of large language model based approaches for information extraction from radiology reports. npj Digit. Med..

[12] Jamaludin A., Kadir T., Zisserman A., McCall I., Williams F.M.K., Lang H., Buchanan E., Urban J.P.G., Fairbank J.C.T. (2023). ISSLS PRIZE in Clinical Science 2023: Comparison of degenerative MRI features of the intervertebral disc between those with and without chronic low back pain. An exploratory study of two large female populations using automated annotation. Eur. Spine J..

[13] Hallinan J.T.P.D., Zhu L., Yang K., Makmur A., Algazwi D.A.R., Thian Y.L., Lau S., Choo Y.S., Eide S.E., Yap Q.V. (2021). Deep learning model for automated detection and classification of central canal, lateral recess, and neural foraminal stenosis at lumbar spine MRI. Radiology.

[14] McSweeney T.P., Tiulpin A., Saarakkala S., Niinimäki J., Windsor R., Jamaludin A., Kadir T., Karppinen J., Määttä J. (2023). External validation of SpineNet, an open-source deep learning model for grading lumbar disk degeneration MRI features, using the Northern Finland Birth Cohort 1966. Spine.

[15] Murto N., Lund T., Kautiainen H., Luoma K., Kerttula L. (2025). Comparison of lumbar disc degeneration grading between deep learning model SpineNet and radiologist: A longitudinal study with a 14-year follow-up. Eur. Spine J..

[16] Nikpasand M., Middendorf J.M., Ella V.A., Jones K.E., Ladd B., Takahashi T., Barocas V.H., Ellingson A.M. (2024). Automated magnetic resonance imaging–based grading of the lumbar intervertebral disc and facet joints. JOR Spine.

[17] Won D., Lee H.J., Lee S.J., Park S.H. (2025). Lumbar spinal stenosis grading in multiple level magnetic resonance imaging using deep convolutional neural networks. Glob. Spine J..

[18] Zwanenburg A., Vallières M., Abdalah M.A., Aerts H.J.W.L., Andrearczyk V., Apte A., Ashrafinia S., Bakas S., Beukinga R.J., Boellaard R. (2020). The Image Biomarker Standardization Initiative: Standardized quantitative radiomics for high-throughput image-based phenotyping. Radiology.

[19] Traverso A., Wee L., Dekker A., Gillies R. (2018). Repeatability and reproducibility of radiomic features: A systematic review. Int. J. Radiat. Oncol. Biol. Phys..

[20] Orlhac F., Lecler A., Savatovski J., Goya-Outi J., Nioche C., Charbonneau F., Ayache N., Frouin F., Duron L., Buvat I. (2021). How can we combat multicenter variability in MR radiomics? Validation of a correction procedure. Eur. Radiol..

[21] Orlhac F., Eertink J.J., Cottereau A.S., Zijlstra J.M., Thieblemont C., Meignan M., Boellaard R., Buvat I. (2022). A guide to ComBat harmonization of imaging biomarkers in multicenter studies. J. Nucl. Med..

[22] Mali S.A., Ibrahim A., Woodruff H.C., Andrearczyk V., Müller H., Primakov S., Salahuddin Z., Chatterjee A., Lambin P. (2021). Making radiomics more reproducible across scanner and imaging protocol variations: A review of harmonization methods. J. Pers. Med..

[23] Mongan J., Moy L., Kahn C.E. (2020). Checklist for Artificial Intelligence in Medical Imaging (CLAIM): A guide for authors and reviewers. Radiol. Artif. Intell..

[24] Nagendran M., Chen Y., Lovejoy C.A., Gordon A.C., Komorowski M., Harvey H., Topol E.J., Ioannidis J.P.A., Collins G.S., Maruthappu M. (2020). Artificial intelligence versus clinicians: Systematic review of design, reporting standards, and claims of deep learning studies. BMJ.

[25] Jorg T., Halfmann M.C., Rölz N., Mager R., Pinto Dos Santos D., Düber C., Mildenberger P., Müller L. (2023). Structured reporting in radiology enables epidemiological analysis through data mining: Urolithiasis as a use case. Abdom. Radiol..

[26] Mariotti F., Agostini A., Borgheresi A., Marchegiani M., Zannotti A., Giacomelli G., Pierpaoli L., Tola E., Galiffa E., Giovagnoni A. (2025). Insights into radiomics: A comprehensive review for beginners. Clin. Transl. Oncol..

[27] Flaiban E., Orhan K., Gonçalves B.C., Lopes S.L.P.D.C., Costa A.L.F. (2025). Radiomics in Action: Multimodal Synergies for Imaging Biomarkers. Bioengineering.

[28] Collins G.S., Moons K.G.M., Dhiman P., Riley R.D., Beam A.L., Van Calster B., Ghassemi M., Liu X., Reitsma J.B., van Smeden M. (2024). TRIPOD+AI statement: Updated guidance for reporting clinical prediction models that use regression or machine learning methods. BMJ.

[29] Fardon D.F., Williams A.L., Dohring E.J., Murtagh F.R., Gabriel Rothman S.L., Sze G.K. (2014). Lumbar disc nomenclature: Version 2.0. Recommendations of the combined task forces of the North American Spine Society, the American Society of Spine Radiology and the American Society of Neuroradiology. Spine J..

[30] Kim H.Y. (2017). Statistical notes for clinical researchers: Chi-squared test and Fisher’s exact test. Restor. Dent. Endod..

[31] Benjamini Y., Hochberg Y. (1995). Controlling the false discovery rate: A practical and powerful approach to multiple testing. J. R. Stat. Soc. Ser. B Methodol..

[32] Cohen J. (1968). Weighted kappa: Nominal scale agreement provision for scaled disagreement or partial credit. Psychol. Bull..

[33] Kehl K.L., Elmarakeby H., Nishino M., Van Allen E.M., Lepisto E.M., Hassett M.J., Johnson B.E., Schrag D. (2019). Assessment of deep natural language processing in ascertaining oncologic outcomes from radiology reports. JAMA Oncol..

[34] Le Guellec B., Lefèvre A., Geay C., Shorten L., Bruge C., Hacein-Bey L., Amouyel P., Pruvo J.P., Kuchcinski G., Hamroun A. (2024). Performance of an open-source large language model in extracting information from free-text radiology reports. Radiol. Artif. Intell..

