# Supplementary material for: Transparent, Reproducible Text-Based Phenotyping of Lumbar Intervertebral Disc Degeneration from 500 Consecutive MRI Reports, with a Pre-Specified Image Analysis Framework
Source: Diagnostics (Basel). 2026 Sep 12;16(18):2951. doi: 10.3390/diagnostics16182951 (PMC13605291; doi:10.3390/diagnostics16182951)
Supplement: Supplementary file 1 [file diagnostics-16-02951-s001.zip › Supplementary_Methods_diagnostics-4483471.pdf]

Supplementary Methods

This document publishes, in auditable form, the rule sets referenced in the main text: the keyword extraction dictionary (Sections 2.3 and 3.3-3.4), the text-derived severity proxy with its precedence and negation rules (Section 2.4), the locked level-mention definition (Section 3.5), the clustering sensitivity analysis code (Section Statistical Handling of Repeated Examinations Within Patients and Supplementary Table S1), and annotated excerpts of the image-analysis pipeline (Section 2.6). Prior to this resubmission, every rule set below was re-executed against the source workbook and verified to regenerate the corresponding published values exactly; the verification points are stated at the end of each section.

S1. Text corpus and preprocessing

For all text-based extraction, the three narrative fields of each report row (morphological keyword [benchmark], source context / level, and primary clinical findings) were concatenated with a field delimiter, lower-cased, and normalized (en dash to hyphen). Empty fields contribute an empty string, so the 49.4% missingness of the structured morphological-keyword field does not remove a report from processing. The same corpus was built identically for all 500 reports; eligibility status was recorded independently of, and prior to, any keyword processing (Section 2.2).

S2. Keyword extraction dictionary (Table 3, Figure 7)

Each of the fifteen descriptor flags is a case-insensitive regular-expression match on the concatenated corpus of Section S1. Word stems are used deliberately so that inflected forms are captured (for example, protru matches protrusion and protruding). Matching is presence/absence per report; repeated mentions do not increment the flag.

Flag	Pattern (regex, case-insensitive)	Descriptor family
`kw_bulge`	`bulg`	disc bulge (incl. diffuse disc bulge, bulging)
`kw_mild`	`mild`	mild severity qualifier
`kw_dehydration`	`dehydrat|desicc`	dehydration / desiccation
`kw_central`	`central`	central location descriptor
`kw_spondylolisthesis`	`spondylolisthesis`	spondylolisthesis
`kw_moderate`	`moderate`	moderate severity qualifier
`kw_protrusion`	`protru`	protrusion (incl. protruding)
`kw_stenosis`	`stenos`	stenosis (canal / foraminal)
`kw_herniation`	`herniat`	herniation (incl. herniated)
`kw_height_loss`	`\bheight loss\b`	disc-height loss (lumbar scope rule; note a)
`kw_extrusion`	`extru`	extrusion (incl. extruded)
`kw_facet`	`facet`	facet-joint descriptor
`kw_foraminal`	`foraminal`	foraminal location descriptor
`kw_severe`	`severe`	severe severity qualifier
`kw_modic`	`modic`	Modic endplate change

Note a (lumbar scope rule). The height-loss flag counts lumbar disc-height loss; the single report describing height loss at the T12 and L1 vertebral bodies (a non-lumbar-disc finding) is not counted.

Verification. Applied to the source workbook, this dictionary regenerates the published included/excluded 2 x 2 tables of all fifteen keywords exactly (Table 3; Figure 7; supplementary association file included_vs_excluded_keyword_fisher_fdr).

S3. Text-derived severity proxy (0-3): dictionary, precedence, and negation rules

The severity proxy is computed on the same concatenated corpus (Section S1). Descriptors are organized into three graded classes, ordered by escalating structural compromise following the consensus lumbar disc nomenclature, version 2.0 (Fardon et al., reference [34] of the main text), with grade 0 as the default:

Grade	Label	Descriptor class
0	none / normal	No degenerative descriptor of grades 1-3 in the report text (default category).
1	mild degenerative change	Contour abnormality without focal displacement: disc bulge.
2	established degenerative change	Focal displacement or degenerative change confined to the disc: protrusion, annular fissure/tear, dehydration/desiccation, disc-height loss.
3	advanced / clinically actionable change	Displacement beyond the disc space or neural compromise: extrusion, sequestration, canal stenosis, nerve-root compression; explicit high-severity qualifiers.

Precedence rule. A report is assigned the highest grade whose descriptor class matches anywhere in its corpus; lower-grade matches are then immaterial. The score is a per-report ordinal proxy for reported severity; the patient-level value is the maximum grade across a patient's reports (Section 2.5).

Negation rule. A matched descriptor is suppressed when it is negated within the same clause, where a clause is the text between sentence or field delimiters ([.;|]) and the negation cues are: no, not, without, free of, negative for, resolved (for example, 'no significant canal stenosis' does not trigger grade 3, and 'no evidence of extrusion' does not trigger grade 3).

Executable locked specification (Python; executed as published; the height-loss family carries the lumbar scope rule of Section S2, note a):

```
NEG = r'(?:\bno\b|\bnot\b|\bwithout\b|\bfree of\b|\bnegative for\b|\bresolved\b)'
HT  = r'(?:(?:loss|reduc)\w*\s+(?:of\s+|in\s+)?(?:normal\s+)?(?:disc\s+)?height'
      r'|height\s+(?:loss|reduc\w*))'
G1 = [r'bulg']
G2 = [r'protru', r'annular\s+(?:tear|fissure)', r'dehydrat', r'desicc', HT]
G3 = [r'extru', r'sequestr', r'canal\s+stenosis',
      r'nerve\s+root\s+compress', r'\bsevere\b', r'\bmarked\b']
def clause_hit(text, pattern):        # clause-scoped, negation-aware match
    for m in re.finditer(pattern, text):
        clause = re.split(r'[.;|]', text[:m.start()])[-1]
        if re.search(NEG, clause): continue
        return True
    return False
grade = 0                             # precedence: highest matching grade wins
if any(clause_hit(t, p) for p in G1): grade = 1
if any(clause_hit(t, p) for p in G2): grade = max(grade, 2)
if any(clause_hit(t, p) for p in G3): grade = max(grade, 3)
```

Scope statement. The category boundaries are consensus-anchored rather than fitted to the data; the assignment of individual synonyms to grades involved author judgment (Section 2.4). All severity statistics in the article are computed under this locked rule; the rule, the analysis code, and the derived per-report severity file are archived and available per the Data Availability Statement, so the mapping can be independently audited or re-specified. The proxy is not a clinical grading instrument and is not equivalent to the Pfirrmann classification; its reference-standard comparison is pre-specified in Section 2.6.1.

Verification. Executed on the source workbook, this locked rule yields the report-level distribution 137 / 137 / 118 / 108 for grades 0-3 (27.4 / 27.4 / 23.6 / 21.6%), the patient-level maximum-severity distribution 100 / 104 / 89 / 93 (n = 386), and the Section 3.6 regression results exactly as published in the revised article.

S4. Locked definition of level mentions (Section 3.5; Figures 8 and 9)

Level mentions are counted as distinct named lumbar interspaces, L1-L2 through L5-S1, after deduplication and expansion of explicit spans. The parser recognizes hyphen, slash, and 'to' separators and shorthand forms (for example, L4/5 and L5/S1); an explicit span (for example, L2-S1) is expanded into its constituent interspaces (L2-L3, L3-L4, L4-L5, L5-S1); repeated mentions of the same interspace

across fields are counted once. The maximum possible value is therefore five. Vertebral-body references (for example, T12) are not level mentions under this definition.

Reference implementation (Python; executed as published):

```
order = ['L1', 'L2', 'L3', 'L4', 'L5', 'S1']
pat = r'l\s*([1-5])\s*(?:-|/|\s+to\s+)\s*(l\s*)?([1-5]|s\s*1)'
def interspaces(text):                    # text: lower-cased corpus of S1
    found = set()
    for m in re.finditer(pat, text):
        a = int(m.group(1)); b = m.group(3).replace(' ', '')
        upper = 'S1' if b == 's1' else 'L' + b
        ai, bi = order.index('L' + str(a)), order.index(upper)
        if bi > ai:                       # expand spans; set() deduplicates
            for i in range(ai, bi):
                found.add(order[i] + '-' + order[i + 1])
    return found                              # |found| is capped at 5 by construction
```

Verification. Applied to the 385 included reports, this parser regenerates the published Section 3.5 values exactly: median 1 (IQR 1-2), mean 1.49 (SD 1.06), range 0-5; distribution 47 / 189 / 92 / 36 / 14 / 7 for 0-5 interspaces; per-interspace counts L4-L5 255 (66.2%), L5-S1 165 (42.9%), L3-L4 103 (26.8%), L2-L3 34 (8.8%), L1-L2 15 (3.9%); and the concordant patient-level prevalences (n = 295). A previously reported token-count summary (mean 2.84, maximum 7), which counted repeated level tokens and vertebral-body references rather than distinct lumbar interspaces, was internally inconsistent and has been removed; all level statistics in the article use the locked definition above.

S5. Clustering sensitivity analyses (Section Statistical Handling of Repeated Examinations Within Patients; Supplementary Table S1)

(a) Cluster-robust re-estimation. For each keyword without a zero cell (eleven of fifteen), the enrichment odds ratio was re-estimated from a logistic model with an independence working correlation and patient-clustered robust (sandwich) standard errors (500 reports in 386 patient clusters), retaining Benjamini-Hochberg correction:

```
m = smf.gee(f'{kw} ~ included', groups='pid', data=df,
            family=sm.families.Binomial(),
            cov_struct=sm.cov_struct.Independence()).fit()  # sandwich SEs
OR = exp(b); CI = exp(b -/+ 1.96*se); q = BH-FDR across the 11 testable keywords
```

Zero-cell keywords (spondylolisthesis, extrusion, foraminal, Modic) have an undefined odds ratio in this model and are assessed by the exact analyses of (b).

(b) Exact one-report-per-patient analyses. All Fisher exact tests were repeated on exactly one report per patient (n = 386), removing clustering by design, under three rules: most recent report per patient, earliest report per patient (rows sorted by patient, report date, and serial number; ties broken by serial number), and 1,000 random draws with a fixed seed (numpy default_rng(42)), with Benjamini-Hochberg correction across all fifteen keywords in each analysis. Full results, including the borderline flag for spondylolisthesis under the earliest-report rule (adjusted p = 0.047; zero-cell keyword), are given in Supplementary Table S1.

(c) Ordinal severity model robustness. The archived script first verifies the locked anchors on the derived per-report file (severity distribution 137/137/118/108; primary model n = 417 with age OR 1.36 per decade, 95% CI 1.19-1.56; female OR 0.97; year OR 1.00) and only then computes: (i) a patient-clustered bootstrap 95% CI for the age odds ratio (2,000 resamples of patients, percentile method, seed 42; result 1.14-1.65); (ii) a patient-level proportional-odds model on each patient's maximum severity (n = 340 patients with a parseable, nonzero age; OR 1.24, 95% CI 1.07-1.44); (iii) the parallel-lines likelihood-ratio test comparing the ordered-logit and multinomial-logit models, with degrees of freedom (J - 2) x p (chi-square 42.83, df = 6, p < 0.001, rejecting the proportional-odds assumption); and (iv) the threshold-specific binary-logit age odds ratios at severity >= 1, >= 2, and >= 3 reported alongside the

primary model and in Supplementary Table S1. Reports with a recorded age of 0 (one report; data-entry artifact) are treated as missing, giving the analytic n of 417 out of 418 parseable ages.

S6. Image-analysis pipeline: annotated code excerpts (Section 2.6)

The pipeline is a single, function-based MATLAB file, lumbarPhase2Pipeline (archived version v2.8; all helper routines are local functions, so the project folder is one deployable artifact). All localizations are fully automatic; the file contains no interactive-input calls of any kind, and the only human involvement is post hoc, case-level visual review of the automatically generated overlays (accept or exclude), with no coordinate input or editing. The excerpts below are lightly abridged verbatim excerpts from the archived file; the complete file, its run-stamped configuration snapshot (CONFIG_used.mat), and the per-run diary log are archived per the Data Availability Statement.

Stage 1 - per-study extraction (processPatientAutomated):

```
info = readPatientDicomVolume(patientPath, CONFIG);   % T2-sagittal series only:
    % orientation cosines (tol 0.15), series regex 'T2|TSE|FSE', >= 5 slices
[midSlice, midIdx, sliceConf, candidateIdx, candidateScores] = ...
    autoSelectMidsagittal(vol, 5);        % symmetry-scored top-5 candidates
for k = 1:numel(candidateIdx)            % retry over candidates
    [tryCenters, tryConf] = autoDetectDiscs(trySlice, CONFIG);
    if size(tryCenters, 1) >= 3, ...; break; end  % accept: >= 3 discs
end
[centers, ord] = sortrows(centers, 2, 'descend');    % caudal -> cranial ordering
names = CONFIG.DISC_NAMES;               % {'L5-S1',...,'L1-L2'}, caudal anchor
saveQcOverlay(midSlice, cAll, names, confAll, patientID, midIdx, ...);
    % automatic per-patient PNG: disc markers + name (confidence) labels +
    % chosen-candidate rank; used only for visual accept/exclude review
[feat, qc] = extractAllDiscFeatures(midSlice, cAll, pxSp, CONFIG);
```

Per-disc feature extraction (extractAllDiscFeatures) computes, for each of the five interspaces: ROI statistics; disc height in pixels and normalized to the adjacent vertebral-body height (estimateDiscAndVbHeight; ratio clipped to the anatomically plausible cap CONFIG.HEIGHT_NORM_CAP = 0.65); a T2 signal index referenced to intracanal cerebrospinal fluid (estimateCsfReference) on a display-normalized image (1st-99th percentile); the nucleus-annulus signal ratio (computeNucleusAnnulusAP); dual-endplate Modic-pattern ratios (computeModicDual); the canal anteroposterior diameter and the posterior disc bulge, converted to millimeters through the DICOM pixel spacing; and a per-disc quality-control flag. Two heuristic per-disc outputs are then derived:

```
% 0..3 severity proxy (mirrors the Phase-1 text scale); additive rule:
if h < 0.20, sc = sc + 2; elseif h < 0.30, sc = sc + 1; end   % height ratio
if s < 0.35, sc = sc + 2; elseif s < 0.55, sc = sc + 1; end   % T2/CSF signal
if nar < CONFIG.NUCLEUS_DARK_RATIO, sc = sc + 1; end          % nucleus/annulus
features.severity_proxy_0_3(d) = min(3, sc);

% explicitly heuristic Pfirrmann-equivalent grade (1..5): hierarchical rule,
% T2 signal first (cutoffs 0.60/0.45/0.30/0.15), refined by height ratio
% (grade IV if h < 0.20; grade V if h < 0.10); unvalidated candidate output
features.pfirrmann_predicted(d) = predictPfirrmann(h, s, ...
    CONFIG.PFIRRMANN_T2_THRESH, CONFIG.PFIRRMANN_HEIGHT_THRESH);
```

Stage 2 - study-level aggregation, merge, and audit (mergeWithExcel):

```
% one row per study (field names as exported):
%   max_severity_0_3, mean_severity_0_3, any_moderate_plus,
%   min/max/mean_disc_height_norm, mean/min_t2_signal_norm,
%   degeneration_burden = mean(1 - disc_height_norm),
%   modic_any, stenosis_any, bulge_any, max_posterior_bulge_mm,
%   max/mean_pfirrmann_predicted, qc_ok, n_discs_detected/valid

% cohort-level calibration before the merge (n >= 30 studies):
%   T2 winsorization to the 1st-99th percentiles;
%   percentile-recalibrated candidate flags (stenosis: 15th pct of the
%   minimum canal diameter; bulge: 75th pct; Modic: 90th pct of
```

```
%   |ratio - 1|), with the fixed-threshold versions preserved in
%   *_fixed columns for sensitivity comparison

R.PatientIDKey  = canonicalPatientId(R.('Patient ID'));   % canonical keys
R.ReportDateKey = canonicalDate(R.('Report Date'));
% same-episode match (CONFIG.MATCH_STRATEGY = 'within_window', 14 d):
%   exact scan-report date match preferred; otherwise the nearest report
%   within 14 days is accepted; a pre-merge dry run reports overlap under
%   all strategies and aborts on zero matches; every merged row carries
%   ImgAuto_match_delta_days (signed) and ImgAuto_match_strategy_used
% post-merge integrity checks verify every ImgAuto_* column survived
```

At the end of a full run, validateAgainstTextLabels computes descriptive imaging-versus-text associations on the merged table (Spearman correlations of the imaging summaries against the keyword flags, included-versus-excluded Wilcoxon group tests, and ROC of the binary imaging flags against the corresponding text flags) and writes them as run-stamped CSV outputs; these are descriptive candidate-level checks against report wording, not the pre-specified expert-reference validation. The gold-standard harness of Section 2.6.1 is implemented in the same file and awaits the reader labels: sampleForPfirrmann builds a severity-stratified labeling template (default 150 studies; per-level Pfirrmann columns plus reader identity, date, and notes), and analyzePfirrmannLabels computes the quadratic-weighted kappa, the 5 x 5 confusion matrix, within-one-grade accuracy, macro F1, and the Spearman correlation of normalized disc height with expert grade, writing the disc-pair table, summary, and confusion matrix as CSV outputs.

S7. Code and data archiving; verification statement

The text-extraction rule sets (Sections S2-S4), the clustering sensitivity scripts (Section S5), and the image-processing pipeline (Section S6) are archived with the study materials and are available from the corresponding author on reasonable request, subject to institutional data-governance approval and a data-use agreement, as stated in the Data Availability Statement. Prior to this resubmission, the rule sets of Sections S2, S4, and S5(a)-(b) were re-executed end-to-end against the source workbook and reproduced the corresponding published values exactly (the fifteen 2 x 2 keyword tables; the Section 3.5 interspace statistics at report and patient level; the Section 3.6 severity distributions and regression results under the locked severity rule of Section S3; and the cluster-robust odds ratios and the one-report-per-patient significance pattern of Supplementary Table S1).
